# Cardiomyocyte p38 MAPKα suppresses a heart–adipose tissue–neutrophil crosstalk in heart failure development

**DOI:** 10.1007/s00395-022-00955-2

**Published:** 2022-10-07

**Authors:** Katharina Bottermann, Lisa Kalfhues, Rianne Nederlof, Anne Hemmers, Lucia M. Leitner, Vici Oenarto, Jana Nemmer, Mirjam Pfeffer, Vidisha Raje, Rene Deenen, Patrick Petzsch, Heba Zabri, Karl Köhrer, Andreas S. Reichert, Maria Grandoch, Jens W. Fischer, Diran Herebian, Johannes Stegbauer, Thurl E. Harris, Axel Gödecke

**Affiliations:** 1 Institute of Cardiovascular Physiology, Medical Faculty and University Hospital Düsseldorf, Heinrich-Heine-University Düsseldorf, Postfach 101007, 40001 Düsseldorf, Germany; 2 Department of Pharmacology, University of Virginia Health System, Charlottesville, VA 22908 USA; 3 Biological and Medical Research Center (BMFZ), Medical Faculty, Heinrich-Heine-University, Universitätsstraße 1, 40225 Duesseldorf, Germany; 4 Institute of Pharmacology, Medical Faculty and University Hospital Düsseldorf, Heinrich-Heine-University Düsseldorf, Postfach 101007, 40001 Düsseldorf, Germany; 5 Institute of Biochemistry and Molecular Biology I, Medical Faculty and University Hospital Düsseldorf, Heinrich-Heine-University Düsseldorf, Postfach 101007, 40001 Düsseldorf, Germany; 6 Institute of Translational Pharmacology, Medical Faculty and University Hospital Düsseldorf, Heinrich-Heine-University Düsseldorf, Postfach 101007, 40001 Düsseldorf, Germany; 7 Department of General Pediatrics, Neonatology and Pediatric Cardiology, Medical Faculty and University Hospital Düsseldorf, Heinrich-Heine-University Düsseldorf, Postfach 101007, 40001 Düsseldorf, Germany; 8 Department of Nephrology, Medical Faculty and University Hospital Düsseldorf, Heinrich-Heine-University Düsseldorf, Postfach 101007, 40001 Düsseldorf, Germany; 9 CARID-Cardiovascular Research Institute Düsseldorf, Medical Faculty, Heinrich-Heine-University, Universitätsstraße 1, 40225 Duesseldorf, Germany; 10 Present Address: Institute of Pharmacology, Medical Faculty and University Hospital Düsseldorf, Heinrich-Heine-University Düsseldorf, Postfach 101007, 40001 Düsseldorf, Germany

**Keywords:** p38 MAPKα, Pressure overload, Lipolysis, Metabolic dysfunction, Cardiac inflammation

## Abstract

Although p38 MAP Kinase α (p38 MAPKα) is generally accepted to play a central role in the cardiac stress response, to date its function in maladaptive cardiac hypertrophy is still not unambiguously defined. To induce a pathological type of cardiac hypertrophy we infused angiotensin II (AngII) for 2 days via osmotic mini pumps in control and tamoxifen-inducible, cardiomyocyte (CM)-specific p38 MAPKα KO mice (iCMp38αKO) and assessed cardiac function by echocardiography, complemented by transcriptomic, histological, and immune cell analysis. AngII treatment after inactivation of p38 MAPKα in CM results in left ventricular (LV) dilatation within 48 h (EDV: BL: 83.8 ± 22.5 µl, 48 h AngII: 109.7 ± 14.6 µl) and an ectopic lipid deposition in cardiomyocytes, reflecting a metabolic dysfunction in pressure overload (PO). This was accompanied by a concerted downregulation of transcripts for oxidative phosphorylation, TCA cycle, and fatty acid metabolism. Cardiac inflammation involving neutrophils, macrophages, B- and T-cells was significantly enhanced. Inhibition of adipose tissue lipolysis by the small molecule inhibitor of *adipocytetriglyceride lipase* (ATGL) Atglistatin reduced cardiac lipid accumulation by 70% and neutrophil infiltration by 30% and went along with an improved cardiac function. Direct targeting of neutrophils by means of anti Ly6G-antibody administration in vivo led to a reduced LV dilation in iCMp38αKO mice and an improved systolic function (EF: 39.27 ± 14%). Thus, adipose tissue lipolysis and CM lipid accumulation augmented cardiac inflammation in iCMp38αKO mice. Neutrophils, in particular, triggered the rapid left ventricular dilatation. We provide the first evidence that p38 MAPKα acts as an essential switch in cardiac adaptation to PO by mitigating metabolic dysfunction and inflammation. Moreover, we identified a heart–adipose tissue–immune cell crosstalk, which might serve as new therapeutic target in cardiac pathologies.

**Supplementary Information:**

The online version contains supplementary material available at 10.1007/s00395-022-00955-2.

## Introduction

Chronic pressure overload (PO) leads to maladaptive cardiac hypertrophy, which is a major risk factor for the development of heart failure. This pathological setting can be mimicked in rodent models by transverse aortic constriction (TAC) or chronic application of angiotensin II (AngII) [1], resulting in hypertrophic cardiomyocyte (CM) growth. However, the pathological type of hypertrophy has a poor prognosis as it possibly progresses to decompensation with gradual decline of cardiac function, left ventricular dilation, and finally to heart failure [58]. Although cardiac hypertrophy and dilation primarily affect the heart itself, the complex events leading eventually to heart failure represent rather a systemic disease. On the molecular level, neuro-humoral stimulation includes sympathetic activation as well as activation of the renin angiotensin system both mediating hypertrophic cardiomyocyte growth and maladaptive ventricular remodeling. Moreover, activation of fibroblasts results in fibrosis [13]. Another hallmark of PO hypertrophy is a sterile inflammation involving the innate and adaptive immune systems. The contribution of macrophages to the progression of hypertrophy development, and the role of T- and B-cells in the transition from hypertrophy to failure is well-established [10]. In contrast, the role of neutrophils in PO hypertrophy remains elusive. Some reports indicated a slight increase in neutrophils during the first days after onset of PO [62], whereas others failed to detect an elevated response by neutrophils leading to the assumption that neutrophil infiltration could be rather a hallmark of ischemic injury than of PO hypertrophy [5]. However, recently this view was challenged by demonstration that neutrophils exacerbated PO induced cardiac dysfunction [61].

p38 MAP kinases (MAPK) belong to the family of stress activated kinases which respond to a variety of extracellular and intracellular signals including cytokines, reactive oxygen species and altered osmotic pressure [32]. Among the four isoforms p38 α, β, γ, and δ, p38 MAPKα is ubiquitously expressed. Activation of p38 MAPK involves a cascade of upstream kinases which mediate phosphorylation of a T–X–Y motif located in the regulatory loop of p38 MAPK [45]. Once activated p38α may divert signaling via phosphorylation of other kinases including MK2/3, MINK and others [26], or it migrates to the nucleus, where multiple transcription factors such as MEF2a/c [15], GATA4 [56], and cMYC [54] are activated by p38α.

In the heart, p38 MAPKα is activated rapidly in response to mechanical stress [55] and G_αq_ mediated signaling [16, 36], as well as in response to pressure overload [12], pacing-induced heart failure [20, 49] and myocardial ischemia [23, 39, 50, 51]. In view of its central function in the cardiac stress response, attempts have been made to elucidate the functional role of p38α by its activation/inactivation in cardiomyocytes. Overexpression of dominant negative mutants of p38 MAPKα led to contradictory findings. Braz et al. showed a progressive CM hypertrophy with and without cardiac stress leading to the assumption that p38α inhibits CM growth via NFAT [4]. In contrast Zhang et al. reported an unchanged cardiac hypertrophy in response to TAC [66] but also reduced apoptosis [65]. The analysis of a constitutive CM specific KO of p38α in response to TAC, however, led to the conclusion that p38 MAPKa mainly promotes cell survival but not CM hypertrophy in response to stress as those mice exhibited increased apoptosis [37]. On the other hand, also the CM-specific activation of p38α activity resulted in cardiomyocyte hypertrophy and apoptosis [33]. It seems clear that p38 MAPKα is involved in cell survival and growth in response to cardiac stress but it remains elusive if this role is more detrimental, as often assumed [24, 63], or rather protective.

Whereas the early studies of cardiomyocyte-specific inactivation of p38 focused on hypertrophy as a cardiomyocyte-specific growth response, more recent work considered also p38α in modulation of intercellular cross-talk during the complex development of cardiac hypertrophy. It was demonstrated that cardiomyocyte p38α was essential to upregulate VEGF expression, which appeared to enhance capillary density in the heart, a response typically associated with pressure overload [46]. This work indicates that CM p38α plays a protective role in heart failure development via paracrine stimulation of angiogenesis and improved perfusion of the myocardium.

To further elucidate the role of p38 MAPKα in myocardial remodeling, we analyzed a mouse line with tamoxifen-inducible p38 MAPKα deficiency in cardiac myocytes and addressed the function of p38 MAPKα in cardiac adaptation to AngII-induced pressure overload. We demonstrate that CM p38 MAPKα acts as a key regulator of myocardial adaptation to PO, by preventing metabolic dysfunction and neutrophil infiltration, which are key events in heart failure development in CM specific p38α deficient mice.

## Experimental procedures

### Animals

All described animal experiments were in accordance with the national guidelines and approved by the local animal care and use committee (LANUV Recklinghausen, Germany, License no AZ: 84-02.04.2014.A220, AZ: 81-02.04.2020.A190) iCMp38αKO mice were generated by crossing C57Bl/6 mice homozygously expressing floxed p38 MAPKα (p38^flox/flox^, Exon 2 and 3) [59] with heterozygous mice expressing the tamoxifen-inducible Cre-recombinase merCremer under control of α-MHC promotor [53, 60]. For KO induction mice received 500 µg 4OH-Tamoxifen (Tx) (5 mg/ml in peanut oil, Sigma #H6278) i.p. for 10 days at 6 weeks age and were allowed to recover from injections for 4 weeks. Animals used for experiments were male mice at the age of 12 weeks. As control, p38^flox/flox^ littermates, equally injected with 4OH-Tx, were used.

Mice received 1.5 mg/kg/d angiotensin II (Sigma, #A9525) via Alzet osmotic mini pumps (model 1003D) for maximum 2 days.

Radiotelemetric blood pressure measurements in conscious mice were performed by implanting pressure-sensing catheters (Data Sciences International, Saint Paul, MN, USA) into the left common carotid artery. After 7 days of recovery and reestablishment of diurnal blood pressure variation, blood pressure measurements were collected continuously with sampling every 20 min for 10-s intervals.

Echocardiographic measurements were performed with Vevo 2100 High Frequency Ultrasound System (Visualsonics, Toronto, Canada) using transducer MS400 (18–38 MHz) as described before [19]. Ejection fraction, end diastolic- and end systolic volume were calculated via Simpson’s protocol from parasternal short and long axis B-Mode images. Left ventricular mass was calculated from left ventricular inner diameter (LVID), left ventricular posterior (LVPW) and anterior wall (LVAW) (diastole) from short axis M-Mode.

For organ harvest mice were sacrificed by cervical dislocation. Hearts were then dissected, the apex of the heart was frozen in liquid nitrogen for RNA and protein analysis, the rest of the heart was OCT embedded and snap frozen in − 40 °C cold isopentane.

The following groups were analyzed: timeline analysis: four different groups of KO and control animals were either sacrificed at baseline (without AngII treatment), or after 12, 24 and 48 h of AngII treatment. Atglistatin treatment: all animals, KO and control, either Atglistatin or vehicle treated, were sacrificed after 48 h of AngII treatment. Propranolol treatment: all animals, KO and control, either propranolol or vehicle treated, were sacrificed after 48 h of AngII treatment. Anti-Ly6G treatment: all animals, KO and control, either anti-Ly6G or isotype treated, were sacrificed after 48 h of AngII treatment.

### Western blot analyses

Proteins were isolated from heart apex in 150 mM NaCl, 10 mM Tris, 0.1% NP 40, pH 7.4, 4 °C using a tissue homogenizer (TissueRuptor, Qiagen). Protein content was measured using *Pierce™ BCA Protein Assay Kit* (Thermo Fisher #23225). SDS-PAGels were loaded with 25–50 μg total protein. Blotting was performed using *Pierce™ G2 Fast Blotter* (Thermo Fisher #62287) with preprogrammed method for mixed molecular weight (25 V, 1.3 mA, 7 min).

Primary antibodies were used in 5% BSA in TBST and are listed in Supplemental Table S1.

Normalization was performed using *REVERT Total Protein Stain* (Li-COR) according to the manufacturer’s recommendation as not otherwise specified.

### Histological analyses

For immunofluorescence staining 8 µm frozen tissue sections were fixed with 4% paraformaldehyde in 0.1 M sodium phosphate buffer pH 7.4 and permeabilized with 0.2% saponin in PBS (137 mM NaCl, 2.7 mM KCl, 8.1 mM Na_2_HPO_4_ × 2H_2_O, 1.76 mM KH_2_PO_4_, pH 7.4). Blocking buffer (10% normal goat serum (NGS) in 0.2% saponin in PBS) was incubated for 60 min, primary antibody (in 2% NGS) overnight, 4 °C, secondary antibody for 3 h at room temperature in the dark. Mounting was performed with *Pro long gold antifade reagent with DAPI* (#P-36935, Invitrogen).

Primary antibodies: *Anti-ADFP antibody* (#ab52356), Abcam, (1:100), *Monoclonal Mouse anti-Ly6g antibody* (#551461), BD Pharming (1:50).

Wheat germ agglutinin: *Lectin from Tritium vulgaris (wheat)* (#W11261), Invitrogen, (10 µg/ml).

Secondary antibodies: *Rhodamine Red-X-AffiniPure Goat Anti-Rat IgG (H* + *L)* (#112-295-167), Jackson Immunoresearch Laboratories, Inc., (1:600), *Cy3-AffiniPure Goat Anti-Rabbit IgG (H* + *L)* (#111-165-144), Jackson Immunoresearch Laboratories, Inc. (1:600). For lipid staining frozen tissue sections were stained after fixation with Bakers formol–calcium fixative (3.6% Formol, 1% CaCl_2_) with filtrated sudan red staining solution (0.3% Sudan Red 7B (#201618, Sigma) in 60% isopropanol).

Tunel Assay was performed using *Click-iT™ Plus TUNEL Assay for in situ Apoptosis Detection, Alexa Fluor™ 488 dye*, ThermoFisher Scientific (C10617) according to the manufacturer’s recommendation.

Microscopy was performed with fluorescence microscope Keyence BZ 9000 (Keyence) or Zeiss Imager.M2. Overviews were taken using the “multi image-function”, where 4× magnifications were automatically put together.

Due to the obvious phenotype of iCMp38αKO mice analysis of histological sections could not be performed in a blinded manner.

### Measurement of lipid profiles

The frozen heart tissue samples (80–120 mg) were homogenized in 2 mL destilled water by precellys ceramic beads using the Minilys homogenizer (Bertin Technologies). For tandem mass spectrometric analysis (Xevo-TQS, Waters) the neutral lipids were extracted from heart tissues by methanol/MTBE (methyl-tert-butylether) procedure (1:5, v,v) [29]. A flow injection analysis method was used and the mobile phases consisted of 50% A: methanol including 50 mM ammonium acetate and 50% B: acetonitrile/2-propanol/chloroform (45:45:10, v,v,v). An internal standard TG 21:1 d5-(17:0/17:1/17:0) was used (Avanti). The analysis of TG’s was performed in the positive ion mode. A neutral loss detection of 8 common acyl fragments in TG’s was performed [40]. The TG species were detected as ammonium adducts [M + NH_4_]^+^. Following mass transitions were used for fatty acids: C16:0 *m*/*z* 273; C16:1 *m*/*z* 271; C18:0 *m*/*z* 301; C18:1 *m*/*z* 299; C18:2 *m*/*z* 297; C18:3 *m*/*z* 295; C20:4 *m*/*z* 321; C22:6 *m*/*z* 345.

### Gene expression profiling

RNA was isolated from the heart apex using *Fibrous Tissue RNeasy Kit*, Qiagen (#74704) according to manufacturer’s recommendation and used for real time-PCR and array and RNA-sequencing analyses.

*Microarray analyses* Total RNA preparations were checked for RNA integrity by Agilent 2100 Bioanalyzer quality control. All samples in this study showed high quality RNA integrity numbers (RIN; > 9). RNA was further analysed by photometric Nanodrop measurement and quantified by fluorometric Qubit RNA assays (Life Technologies).

Synthesis of cDNA and subsequent fluorescent labelling of cRNA was performed according to the manufacturers’ protocol (One-Color Microarray-Based Gene Expression Analysis/Low Input Quick Amp Labeling; Agilent Technologies). Briefly, 100 ng of total RNA were converted to cDNA, followed by in vitro transcription and incorporation of Cy3-CTP into nascent cRNA. After fragmentation labelled cRNA was hybridized to *Agilent SurePrint G3 Mouse GE 8* × *60K Microarrays* for 17 h at 65 °C and scanned as described in the manufacturers’ protocol.

Signal intensities on 20 bit tiff images were calculated by Feature Extraction software (FE, Vers. 11.0.1.1; Agilent Technologies). Data analyses were conducted with *GeneSpring GX* software (Vers. 12.5; Agilent Technologies). Probe signal intensities were quantile normalized across all samples to reduce inter-array variability [2]. Input data pre-processing was concluded by baseline transformation to the median of all samples.

To improve signal-to-noise ratio, a given transcript had to be expressed above background (i.e., called “detected” by FE) in all four replicates in any one of two, or both conditions to be further analysed in pairwise comparisons. Differential gene expression was statistically determined by moderated *T* tests (Benjamin–Hochberg FDR corrected, *p*(corr) < 0.05).

Microarray data were analysed using the *IPA Ingenuity Pathway Analysis software Package* (Qiagen Inc. 2016). The data set was subject to core analysis applying “Experimentally observed” as analysis filter. Canonical pathways, upstream regulators and causal networks tools were used to identify affected regulatory networks.

*RNA-Seq analyses* For transcriptome analyses total RNA samples were quantified using *Qubit RNA HS Assay* (Thermo Fisher Scientific). Quality was measured by capillary electrophoresis using the *Fragment Analyzer* and the ‘*Total RNA Standard Sensitivity Assay’* (Agilent Technologies, Inc. Santa Clara, USA). All samples in this study showed high quality RNA Quality Numbers (RQN; mean = 9.0). Library preparation was performed with the ‘*VAHTS™ Stranded mRNA-Seq Library Prep Kit’ for Illumina*^*®*^ according to the manufacturer’s recommendations. 850 ng total RNA were used for mRNA capturing, fragmentation, the synthesis of cDNA, adapter ligation and library amplification. Bead purified libraries were normalized and finally sequenced on the NextSeq 550 system (Illumina Inc. San Diego, USA) with a read setup of 1 × 75 bp. The bcl2fastq tool was used to convert the bcl files to fastq files as well as for adapter trimming and demultiplexing.

Data analyses on fastq files were performed with CLC Genomics Workbench (version 21.0.4, QIAGEN, Venlo, NL). The reads of all probes were adapter trimmed (Illumina TruSeq) and quality trimmed (using the default parameters: bases below Q13 were trimmed from the end of the reads, ambiguous nucleotides maximal 2). Mapping was done against the Mus musculus (mm10; GRCm38.86; March 24, 2017) genome sequence. After grouping of samples (three biological replicates each) according to their respective experimental condition, a differential expression analysis of the two groups was made and statistically determined using the Wald test. The statistics are based on the fit of a Generalized Linear Model with a negative binomial distribution. The resulting *P* values were corrected for multiple testing by FDR and Bonferroni-correction. A *p* value of ≤ 0.05 was considered significant.

*Quantitative real time PCR* 1 µg mRNA was transcribed into cDNA using *QuantiTect Reverse Transcription Kit*, Qiagen (#205313). Real-time quantitative PCR (qPCR) was performed using *Maxima SYRB Green/ROX qPCR Master Mix*, Thermo Scientific (#K0223) and *StepOne Plus Real-Time PCR Detection System* (Applied Biosystems). All genes were amplified with 1 cycle at 95 °C for 10 min followed by 40 cycles at 95 °C for 15 s and 60 °C for 60 s. The specificity of amplicons was verified by melting curve analysis starting with 95 °C for 15 s, then 60 °C for 60 s. Thereafter the temperature was increased 0.3 °C every 15 s, until the final temperature of 90 °C was reached. Two technical replicates of every biological replicate (*n* = 4/group) were used for qPCR analysis.

Because other commonly used internal reference genes as *Hprt* or *Gapdh* can be subject to regulation under various pathological conditions [34], we screened microarray data for stably expressed genes. *Nudc* showed lowest expression differences under all conditions compared by microarray analysis which was further validated by qPCR and was, therefore, used as reference gene. Due to variable expression *Hprt* and *Gapdh* had to be excluded.

Specific gene expression was first normalized to *Nudc* and then compared with control groups using both the comparative Cq method [25] as well as *X*(0) method. There the exponentially related *C*_t_ values are converted into linearly related *X*(0) values, where *X*(0) represent the amount of starting material in a qPCR experiment [48, 57]. Relative measurement for the following transcripts was performed: *Il6*, *Il1b*, *Cxcl5*, *Cxcr2*, *Slc2a4*, *Ppargc1a, PDK4, Angptl4, TGFb2* (*KiCqStart*^*®*^* SYBR*^*®*^* Green Primers*, Sigma (#KSPQ12012)), for primer sequence see Supplemental Table S2.

To compare different qPCR plates a consistent calibrator was included on every plate. The calibrator was made of mRNA from hearts of different animals as well as different conditions. The mRNA was pooled and transcribed into cDNA, to guarantee that the same cDNA was used for each plate.

### Transmission electron microscopy

Mouse hearts were prepared immediately after sacrifice by cervical dislocation and pre-perfused by aortic cannulation and retrograde perfusion with ~ 10 ml of Krebs–Henseleit-Buffer (118 mM NaCl, 4.7 mM KCl, 1.2 mM MgSO_4_∙7H_2_O; 1.2 mM KH_2_PO_4_; 25 mM NaHCO_3_; 8,32 mM glucose; 1 mM lactate; 0.1 mM pyruvate; pH 7.4). After perfusion with 20 ml fixation buffer (0.1 M sodium cacodylate buffer pH 7.4; 1.2% glutaraldehyde, 1% paraformaldehyde), hearts were cut at respective regions to obtain tissue blocks (size 1 × 1 × 2 mm) and stored in storage buffer (0.1 M sodium cacodylate buffer pH 7.4; 1% glutaraldehyde). Samples were stained with 1% osmium tetroxide for 50 min and with 1% uranyl acetate/1% phosphotungstic acid for 1 h. A graded acetone series was used for dehydration before samples were embedded in spur epoxy resin at 65 °C for 24 h. Ultrathin sections were prepared using a microtome and images were acquired using a standard transmission electron microscope (Hitachi, H600) at 75 V equipped with Bioscan model 792 Gatan camera.

### Atglistatin treatment

The lipolysis inhibition study was performed using Atglistatin, a small molecule inhibitor of adipose triglyceride lipase (ATGL) [30]. Atglistatin (Sigma-Aldrich, TargetMol) was administered via food at a dose of 0.4 mg/g CHOW food [52], 2 days prior to AngII mini-osmotic pump implantation and continued for 48 h thereafter. Mice were starved for 4–5 h prior to sacrifice. Plasma glycerol was measured using a commercial kit (Sigma-Aldrich, #MAK117), and performed according to the manufacturer's recommendation.

### Propranolol treatment

Propranolol (Sigma-Aldrich) was solved in phosphate buffered saline (pH 5) and administered via drinking water (10 mg/kg/day) for 24 h and afterward for 48 h together with AngII via osmotic minipumps (10 mg/kg/day). Efficacy of propranolol was proven by a reduction in heart rate compared to vehicle-treated animals after dobutamine stimulation (1 mg/kg BW) (Supplemental Fig. S5A).

### Flow cytometric analysis of hearts

Cells were isolated from the heart by a protocol adapted from [18, 43]. Hearts were isolated and flushed with PBS/heparin to remove the blood. Atria and valves were removed and hearts were cut in ~ 1 mm pieces using a tissue chopper (McIlwain tissue chopper, Cavey Laboratory Engineering Co. Ltd.). Tissue was incubated at 37 °C for 45 min in 450 U/mL collagenase type I (Worthington #LS004197) and 60 U/mL DNAse I (Roche Diagnostics #10104159001) in HBSS (Gibco Life Technologies #14025). After 15- and 30-min incubation tissue clusters were triturated by pipetting 12 times using a 10 mL serological pipette. At the end of the 45 min incubation period final trituration was performed by pipetting 30 times with a 1 mL pipette and the cells were centrifuged at 300×*g* for 10 min. The pellet was resuspended in FACS buffer (PBS with 0.5% BSA and 2 mM EDTA) and filtered over 100 and 40 μm filters. Cardiomyocytes were removed by 1 min centrifugation at 50×*g*. The supernatant was centrifuged 10 min at 300×*g* and the pellet was dissolved in PBS and incubated with Fc-block (Biolegend, #101302) and, when applicable, Fixable viability dye eFluor 780 (eBioscience #65-0865-14). After washing, cells were stained in FACS buffer with the antibodies listed in Supplemental Table S3. All cells were washed prior to acquisition on *BD FACSCanto II*. Data were analysed using *BD FACSDiva software version 8.0.2*. A gating scheme is depicted in Supplemental Fig. S4.

### Granulocyte depletion

Neutrophil granulocytes were depleted by i.p. injection of 500 μg anti-Ly6G-antibody, clone 1A8 (BioXCell, #BE0075-1) and corresponding isotype control (InVivoMab Rat IgG2a, clone 2A3, BioXCell, #BE0089) 16 h prior to osmotic mini pump implantation.

For flow cytometric analysis of circulating neutrophils EDTA anticoagulated blood was collected 48 h after implantation of osmotic mini pumps and stained followed by lysis of red blood cells. Cells were fixed in 1% neutral buffered paraformaldehyde for 20 min at room temperature, according to a protocol by [6]. To prevent unspecific binding, cells were incubated with anti-CD16/32 antibody (clone 93; Biolegend, San Diego, CA, USA) before antibody staining.

CD11b-PE (M1/70, BD Biosciences, San Jose, CA, USA) and anti-Ly-6C-AlexaFluor^®^ 488 (HK1.4) were used to detect neutrophils and monocytes [21]. Respective isotype control IgGs were used to set all flow cytometric gates. Measurement and data analysis were performed using a Gallios™ Flow Cytometer, and Kaluza^®^ Flow Analysis Software (both Beckman Coulter Inc., Krefeld, Germany).

### Statistics

All data are presented as mean ± standard deviation. Data of each parameter were directly compared between control and iCMp38αKO mice. Statistical analysis was performed using Graph Pad Prism 5 and 8. Repeated measurements with two variables (time and genotype) were analyzed by two-way ANOVA or mixed-effects model in case of missing values followed by Bonferroni’s multiple comparisons test to compare the genotypes at each timepoint. Two groups were compared with unpaired, two tailed *t* test. For comparison of more than two groups, one-way ANOVA followed by Tukey’s multiple comparisons test was used as not otherwise specified. The present study must be classified as an exploratory study and all calculated *p* values are descriptive.

The following groups were compared when treatment was applied (Atglistatin, Propranolol, anti-Ly6G): ctrl. vehicle/IT treated vs. KO vehicle/IT treated, ctrl vehicle/IT treated vs. ctrl treated, ctrl treated vs. KO treated, KO vehicle/IT treated vs. KO treated. Inter-group comparisons were specified post-hoc. Correlation between cardiac neutrophil levels and ejection fraction (EF) was tested by Pearson correlation.

The sample sizes for the functional analysis were based on a power calculation and modified in line with the 3R principles and according to the experimental needs.

Statistical outliers were identified via ROUT test, *Q* = 1%. Based on this analysis two animals were excluded from the analysis in the following experiments: granulocyte depletion: KO, IT treated for parameter ejection fraction. Atglistatin treatment: KO, Atglistatin treated for parameter neutrophil number.

## Results

### p38 MAPKα is protective during the early phase of cardiac adaptation to pressure overload

**Fig. 1:**
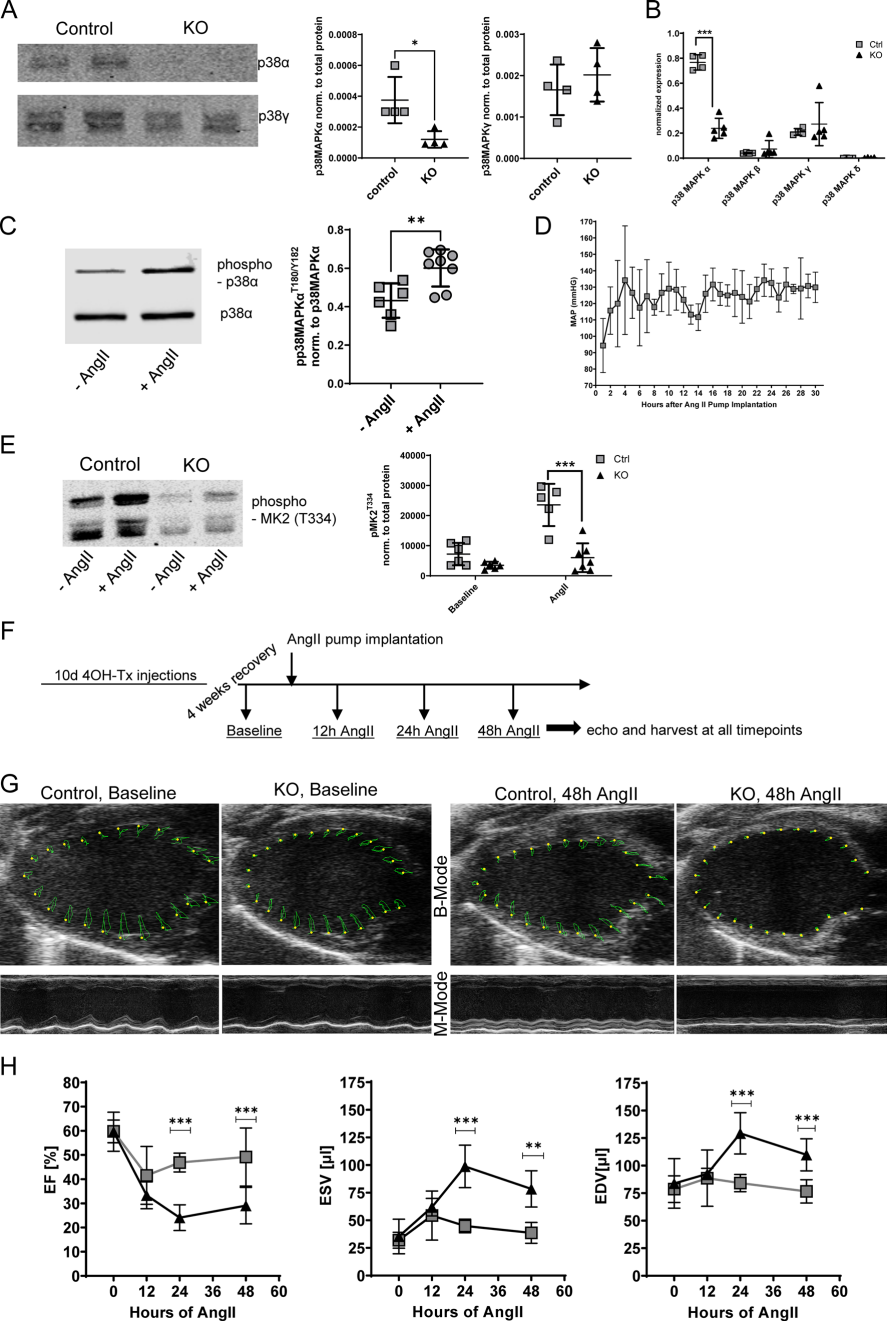
Verification of knockout and cardiac function. **A** Protein expression of p38 MAPKα and p38 MAPKγ in cardiac tissue of control and iCMp38αKO animals. *n* = 4, data represent mean ± SD, unpaired, two-tailed *t* test, *p < 0.05. **B** Transcript expression of p38 MAPK isoforms in whole heart tissue normalized to *Nudc.* *n* = 4–5, data represent mean ± SD, unpaired, two-tailed *t* test, ***p < 0.001. **C** Phosphorylation of cardiac p38 MAPKα after 48 h AngII stimulation compared to unstimulated animals. Phospho values were normalized to total p38 MAPKα signal. *n* = 6–8, data represent mean ± SD, unpaired, two-tailed *t* test, **p = 0.006. **D** Mean arterial pressure measured in the first 30 h after AngII pump implantation in control mice. *n* = 5, data represent mean ± SD. **E** Phosphorylation of p38 MAPKα target MK2 at T334 in control and KO animals with and without AngII stimulation. AngII was applied for 48 h. *n* = 5–7, data represent mean ± SD, mixed-effects analysis. Bonferroni’s multiple comparisons test was used to compared control vs. KO at each timepoint, reported are *p* values below 0.05, ***p < 0.001. **F** Schematic of experimental outline for analysis of cardiac function. **G** B- and M-Mode images of parasternal long axes of control and iCMp38αKO hearts at baseline (left panel) and after 2d of AngII treatment (right panel). B-Mode images show wall displacement. **H** Ejection fraction (EF), end diastolic (EDV) and end systolic volume (ESV) of control and iCMp38αKO hearts at baseline and after 12, 24 and 48 h of AngII treatment. Control: grey square, KO: black triangle, *n* = 5–17, Data represent mean ± SD, two-way ANOVA followed by Bonferroni’s multiple comparisons test was used to compare control vs. KO at each timepoint, reported are *p* values below 0.05, **p < 0.01, ***p < 0.001

To study the role of p38 MAPKα in response to PO, iCMp38αKO mice were generated by cross breeding p38 MAPKα^flox/flox^ mice [59] with aMHC mercremer deleter mice [53]. Activation of the cre recombinase by hydroxytamoxifen following a well-established protocol [17] led to a successful elimination of p38 MAPKα in hearts (Fig. 1A). Other isoforms of p38 MAPK were not affected by KO of the α isoform (Fig. 1A, B). Administration of angiotensin II (AngII) via osmotic minipumps activated p38 MAPKα and increased mean arterial pressure in control mice (Fig. 1C, D). Western blot analysis of phosphorylation of the known p38 MAPKα target MAPKAP kinase 2 (MK2) showed a reduced phosphorylation at T334 in response to 48 h AngII stimulation in KO hearts compared to control hearts (Fig. 1E). Baseline analysis of cardiac function of iCMp38αKO hearts via echocardiography revealed no differences in comparison with control mice (Fig. 1G, H, Supplemental Table S4). However, after challenging the animals by administration of AngII to increase cardiac afterload iCMp38αKO hearts developed a highly impaired systolic pump function (EF: 29 ± 7.6%) as well as an extensive left ventricular dilation (EDV: 109.7 ± 14.6 µl, ESV: 78.4 ± 16.5 µl) within 48 h after onset of AngII treatment. (Fig. 1G, H, Supplemental Table S4). As merCremer deleter mice did not show similar changes in response to 48 h of AngII treatment, this phenotype could be clearly assigned to KO of p38 MAPKα (Supplemental Fig. S1). To further investigate the fast development of the dilative cardiomyopathy in iCMp38αKO hearts we performed additional analysis of cardiac function at 12 and 24 h (Fig. 1H). After 12 h of AngII treatment, control as well as iCMp38αKO mice showed a reduced systolic pump function indicated by a drop of EF to 41.5 ± 12% (control) and 33.3 ± 5.6% (KO), respectively. A major dilation of the left ventricles was not observed. Control mice started to adapt during the next 12 h and increased their EF. In contrast, hearts of iCMp38αKO mice failed to adapt and started to dilate, which was evident from the further decrease in EF and an increase in EDV and ESV. After 48 h, EF had almost returned to basal levels in controls but remained low in iCMp38αKO mice.

### Cardiac gene expression profile reveals downregulation of metabolic genes

**Fig.2:**
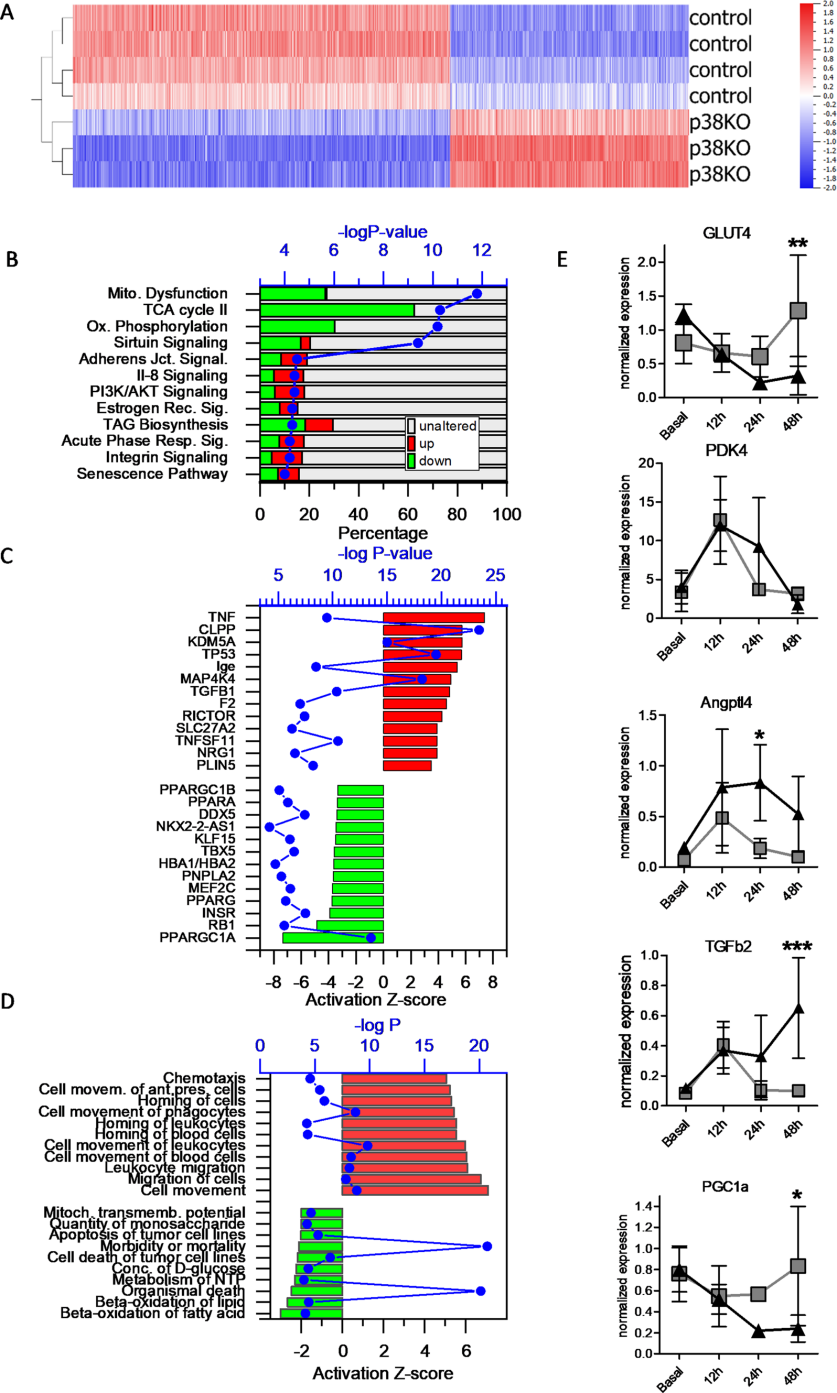
Transcriptomic analysis of gene in control and iCMp38αKO hearts after 2 days of AngII treatment, *n* = 3–4. **A** Hierarchical cluster analysis of differentially expressed genes (FC ≥ 1.5, *p* < 0.01). **B** Canonical pathways identified by ingenuity pathway analysis (IPA) of differentially expressed genes. The top 12 pathways are shown. Stacked bars represent percentages of regulated genes of all genes assembled under the specified term (red: upregulated; green: downregulated; gray unaltered). Blue: − log *p* value of regulation. **C** IPA analysis of upstream regulators. Upstream regulators with an absolute activation *Z*-score ≥ 4, − log*P* ≤ 4 are shown. **D** IPA analysis of altered “Diseases and Functions”. The top activated (red) and suppressed (green) functional terms (absolute activation *Z*-score ≥ 2, − log*P* ≤ 4) are listed. **E** Kinetic analysis of gene expression for selected genes as indicated. Data show the expression levels for control (gray squares) and iCMp38KO hearts (black triangles) over the indicated time course normalized to *Nudc* (*n* = 4 each data point). Data represent mean ± SD. Two-way ANOVA followed by Bonferroni’s multiple comparisons test was used to compare control vs. KO at each timepoint, reported are *p* values below 0.05, *p < 0.05, **p < 0.01, ***p < 0.001)

To identify molecular alterations explaining this severe phenotype, transcriptomic analysis of the control and iCMp38αKO hearts was performed after 12 and 48 h of AngII stimulation. Hierarchical clustering of the gene expression data obtained after 48 h AngII clearly separated control from iCMp38αKO hearts. In total, 3433 genes were statistically significantly changed (*p* < 0.01, *q* < 0.11, fold change > 1.5) (Fig. 2A). Ingenuity pathway analysis (IPA) performed on the differentially expressed genes revealed that genes assembled under the terms “mitochondrial dysfunction”, “oxidative phosphorylation” and “TCA cycle” were substantially altered. Among these terms, “oxidative phosphorylation” had the highest negative *Z*-score (− 7.1). In general, affected genes which were summarized under these terms, were consistently downregulated (Fig. 2B). Another enriched pathway was related to sirtuin signaling. This term had a high *p* value for the gene enrichment, but the *Z* value of 1.6 revealed that there was not a clear direction of regulation. Since sirtuins also affect the expression of mitochondrial genes, the overlap with the term mitochondrial dysfunction may explain why this term was found to be enriched.

Upstream regulator analysis implemented in the IPA software package identified possible regulators, which might mediate the observed gene expression changes. High activation *Z*-scores (> 4) indicated that pro-inflammatory factors (*Tnfα, Tnfsf11*), proteins involved in lipid metabolism (*Slc27A2, Plin5*), and regulators of gene expression (*Kdm5a, Tp53*) were upregulated. Among the regulators which were assumed to be downregulated, *Pgc1α* (*Ppargc1*) was the top candidate which might modify the observed differences between control and iCMp38αKO hearts (*p* value of overlap 3 × 10^–14^, activation *Z*-score − 7) (Fig. 2C). Moreover, the related transcription factors (*Pgc1β, Pparα, Pparγ*) were also listed among the top downregulated upstream regulators. Of note, *Pgc1α* by itself was down regulated which underlines the possible involvement of *Pgc1α* in setting the cardiac phenotype of iCMp38αKO hearts. Interestingly, *Kdm5a, Mapk4k, Tgfb1, Rictor, Ppargc1a, Insr,* and *Rb1,* which were among the top upstream regulators which might be involved the dilative phenotype of iCMp38αKO hearts were also found with comparable Z-scores in a proteomic analysis of human heart biopsies from patients with arrhythmogenic right ventricular cardiomyopathy (ARVC) and dilative cardiomyopathy (DCM) [9].

Analysis of the transcriptomic data by the “Diseases and Functions” IPA tool in Fig. 2D revealed that the top upregulated biological processes (highest activation *Z*-score) were all related to chemotaxis, leukocyte migration, and homing of leukocytes, suggesting that the phenotype of iCMp38αKO mice involved inflammatory processes. The lowest *Z*-scores had the functional terms “oxidation of fatty acids/β oxidation” which is in line with downregulation of mitochondrial function identified by the “canonical pathway” function (Fig. 2B). In view of a possibly compromised metabolism, the time course of expression for a few selected genes which were related to mitochondrial biogenesis (*Pgc1α*), lipid and glucose metabolism (*Glut4, Angptl4, Pdk4*) or fibrosis (*Tgfb2*) and which were found to be differentially expressed in the microarray, was analyzed (0, 12, 24, 48 h post AngII application). Following mRNA levels of *Pgc1α* over time confirmed a statistically significant downregulation after 48 h AngII treatment in comparison with baseline and to control hearts (Fig. 2E). Moreover, *Tgfb2* and *Angptl4* were upregulated, the insulin sensitive glucose transporter *Glut4* was downregulated in iCMp38αKO hearts in response to AngII stimulation. *Pdk4* showed only a transient increase, which was found in control and iCMp38αKO hearts.

In addition, we performed transcriptomic analysis after 12 h of AngII treatment to uncover early changes in gene expression which might give insight into causative mechanisms leading to the heart failure phenotype. Using a significance level of *p* < 0.05, absolute fold change > 1.3 led to the identification of 239 differentially expressed transcripts. Upon hierarchical clustering, control and iCMp38KO transcriptomes were clearly separated (Supplemental Fig. S2). Pathway analysis revealed only 3 altered pathways with an activation *Z*-score > 2. Interestingly, “oxidative phosphorylation” which belonged to the most prominent pathways altered after 48 h AngII treatment was also found in the early transcriptome (*Z*-score − 3.32, *p* < 10^–7^), although only a minor fraction of transcripts assembled under this term was differentially expressed.

### Lipid accumulation and neutrophil infiltration in iCMp38αKO hearts in response to AngII

**Fig. 3:**
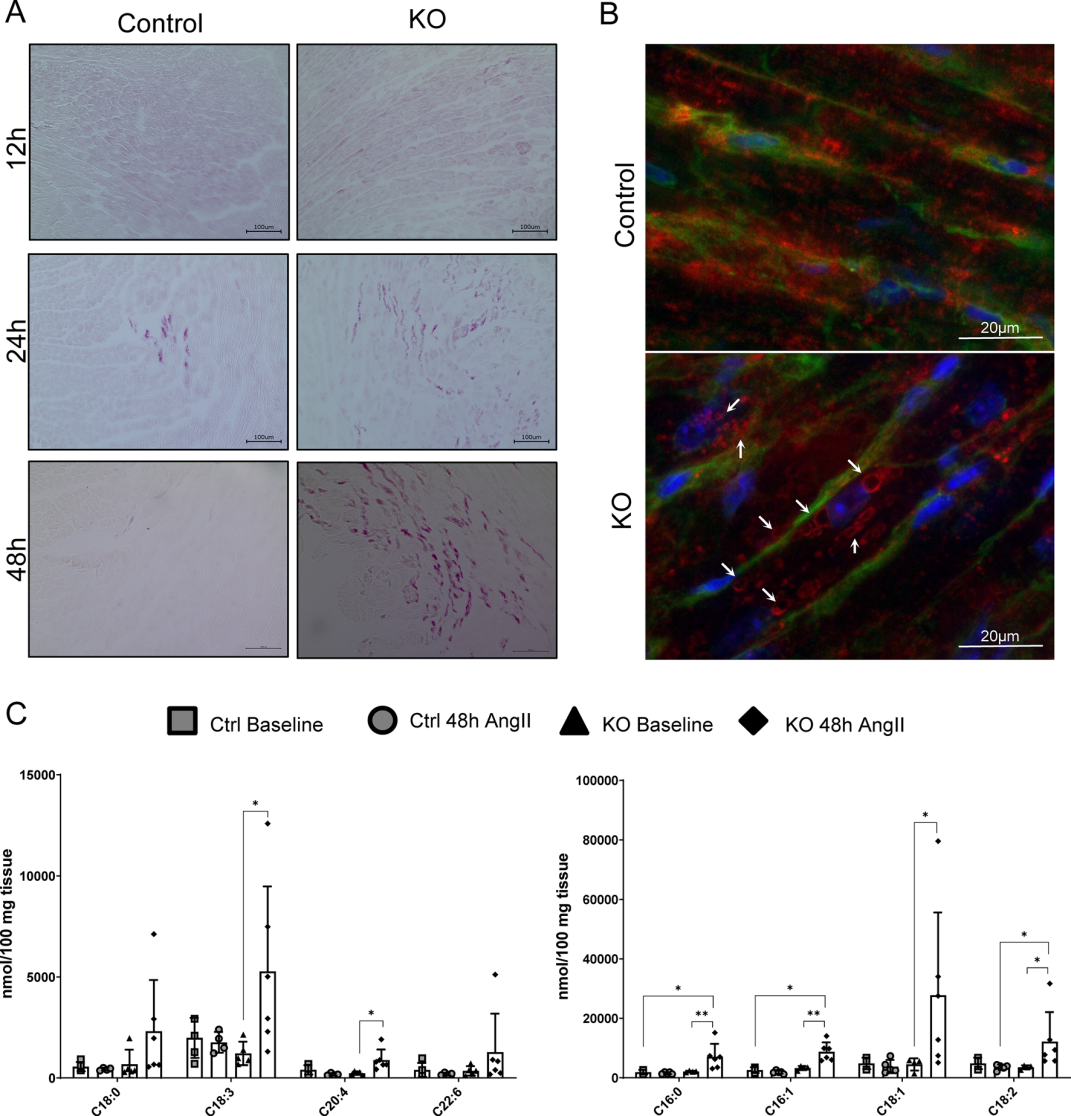
Time course analysis of lipid accumulation. **A** Representative magnifications of hearts after sudan red 7B staining of control and iCMp38αKO hearts after 12, 24 and 48 h of AngII treatment. *n* = 5–7. **B** Anti-perilipin 2 immunofluorescence staining of control and iCMp38αKO hearts after 48 h of angiotensin II treatment. Red: perilipin 2, green: wheat germ agglutinin (WGA), blue: DAPI. Arrows point to lipid droplets. **C** Fatty acid profile of control and iCMp38αKO hearts at baseline and after 48 h of AngII treatment. *n* = 4–6. Kruskal–Wallis test with Dunn’s multiple comparisons test was used to compare each group to another. Reported are *p* values below 0.05. *p < 0.05, **p < 0.01, ***p < 0.001

In line with the results from gene expression, histological analysis revealed a progressive lipid accumulation (Fig. 3A) in iCMp38αKO hearts, which increased concomitantly with the decline of cardiac function. After 12 h of AngII treatment a disseminated, disperse lipid accumulation occurred in hearts of control and iCMp38αKO mice. After 24 h, lipid accumulation was elevated in iCMp38αKO hearts and appeared as droplets in large areas of the heart, while control hearts showed only minor spots of lipid droplet accumulation. After 48 h lipid accumulation further declined in control hearts, while lipids were still high in iCMp38αKO hearts (Fig. 3A). Immunofluorescence staining of the lipid droplet coating protein perilipin 2 in control and iCMp38αKO hearts after 48 h AngII demonstrated the appearance of vesicular structures characteristic for lipid droplets (Fig. 3B). In line with these findings, measurement of fatty acid profiles before and after AngII stimulation revealed elevated levels for all measured fatty acids in iCMp38αKO hearts after 48 h AngII treatment (Fig. 3C). Electron microscopy of heart tissue demonstrated a normal mitochondrial structure in iCMp38αKO hearts despite the coordinated downregulation of mitochondrial genes (Supplemental Fig. S3).

**Fig. 4:**
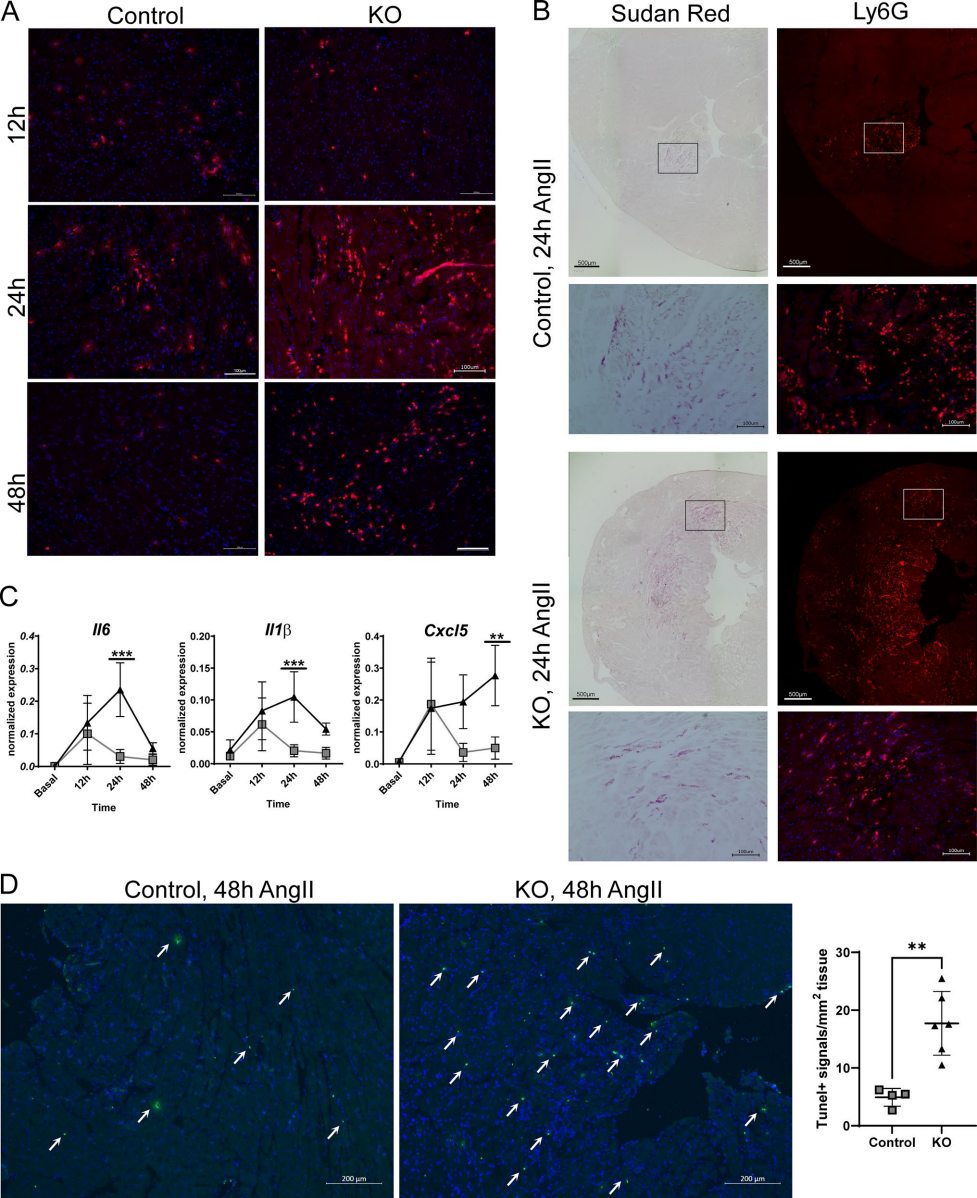
Time course analysis of inflammation. **A** Representative magnifications of hearts after anti-Ly6G immunofluorescence staining of control and iCMp38αKO hearts after 12, 24 and 48 h of AngII treatment. *n* = 5–7. **B** Colocalization of lipid accumulation and neutrophil infiltration in control and iCMp38αKO mice after 24 h AngII treatment. **C** Gene expression level of *Il6, Il1β* and *Cxcl5* over time course in control and iCMp38αKO hearts. *n* = 4. Data represent mean ± SD. Statistical analysis was performed using two-way ANOVA followed by Bonferroni’s multiple comparisons test to compare control vs. KO at each timepoint. Reported are *p* values below 0.05. *p < 0.05, **p < 0.01, ***p < 0.001. **E** TUNEL positive nuclei (arrows) of control and iCMp38αKO hearts after 48 h of AngII treatment and corresponding quantification. *n* = 4–6. Data are presented as mean ± SD. Statistical analysis was performed using unpaired, two-tailed *t* test. **p < 0.01

Since transcriptomic data indicated an elevated inflammation in iCMp38αKO hearts, we investigated if inflammation was associated with lipid accumulation. Anti-Ly6G immunostaining revealed an increasing infiltration of iCMp38αKO hearts by neutrophil granulocytes. At 12 h of AngII stimulation minor neutrophil infiltration was observed, which substantially expanded at 24 h, and stayed high at 48 h (Fig. 4A). Of note, neutrophils mainly appeared in tissue areas with lipid accumulation, suggesting a link between pro-inflammatory signals and lipid droplet formation in cardiac myocytes (Fig. 4B). In control hearts, however, neutrophils only appeared as minor spots (Fig. 4A) and returned to basal levels after 48 h. Similar to the transient infiltration of neutrophils in control hearts, the expression levels of pro inflammatory cytokines and chemokines such as *Il6*, *Il1-β* and *Cxcl5* were only transiently enhanced (12 h AngII stimulation) and then returned to basal levels. In contrast, iCMp38αKO hearts revealed a similar increase at 12 h and an even higher upregulation at 24 h. Then, *Il6* and *Il1-β* levels declined at 48 h, whereas the chemoattractant for neutrophils, *Cxcl5*, further increased (Fig. 4C). In addition, we observed a slightly increased number of apoptotic cells (Tunel assay) after 48 h of AngII treatment (Fig. 4D).

### Inhibition of lipolysis reduces neutrophil infiltration and improves cardiac function

**Fig. 5:**
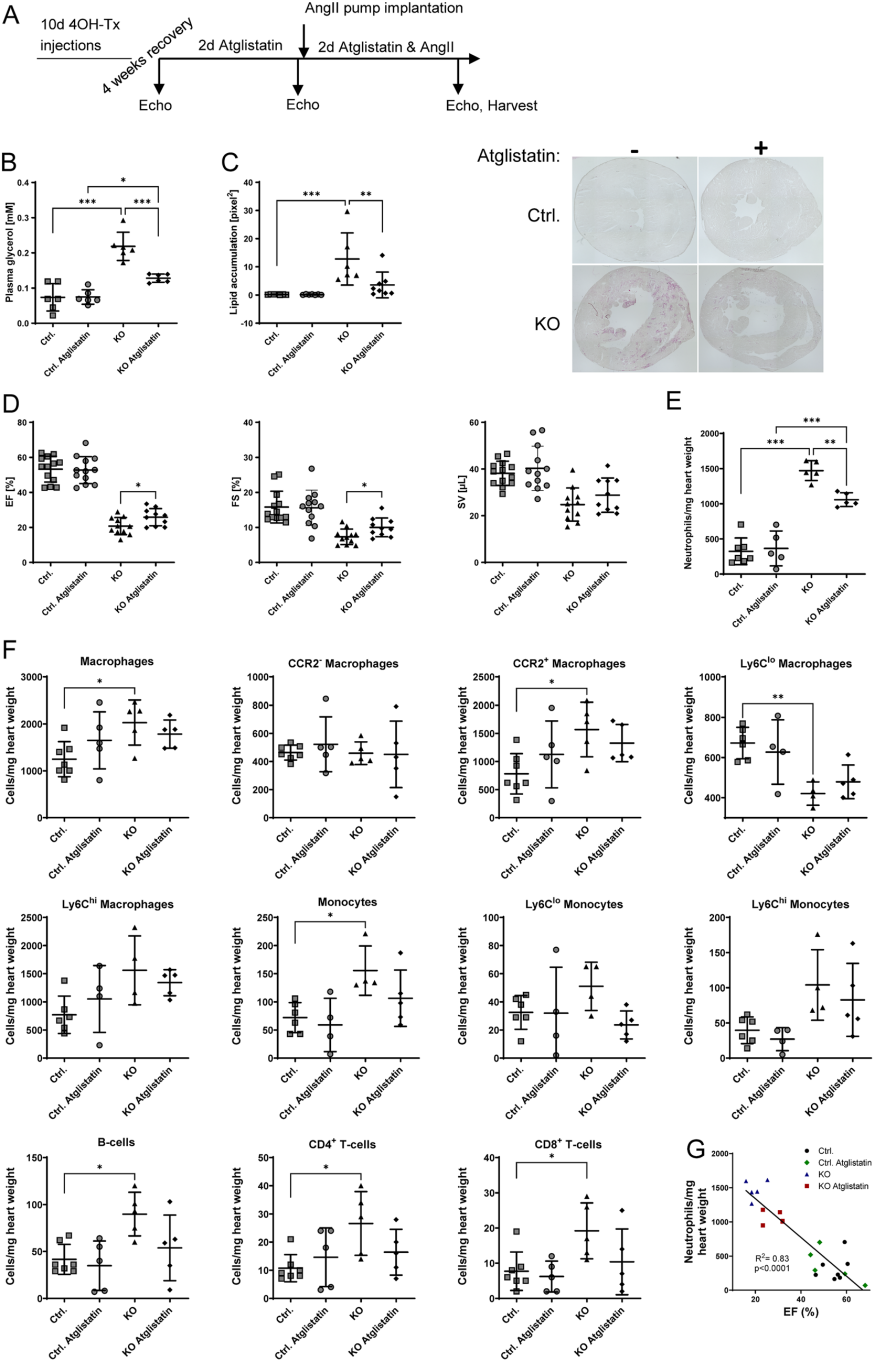
Atglistatin treatment: **A** schematic of the experimental outline. **B** Plasma glycerol level of control and iCMp38αKO mice (*n* = 6). **C** Representative images of sudan red 7B staining and quantification of lipid accumulation of these images of control and iCMp38αKO hearts (*n* = 6–8). **D** EF, fractional shortening (FS) and stroke volume (SV) of control and iCMp38αKO hearts. *n* = 10–13, unpaired, two-tailed *t* test was used to compare KO vs. KO Atglistatin. **E** Flow cytometric analysis of cardiac neutrophils (CD11b^+^Ly6G^+^). **F** Flow cytometric analysis of cardiac immune cells (total macrophages (CD11b^+^Ly6G^−^CD64^+^), CCR2^−^, CCR2^+^, Ly6C^lo^ and Ly6C^hi^ macrophages, total monocytes (CD11b^+^Ly6G^−^CD64^−^MHCII^−^), Ly6C^lo^ and Ly6C^hi^ monocytes, B-cells (CD45^+^CD19^+^CD3^−^) and CD4^+^- and CD8^+^ T-cells (CD45^+^CD3^+^CD19^−^)) (*n* = 4–7). G) Correlation between cardiac neutrophil number and EF. All data shown are from animals after 48 h of AngII treatment either with or without pretreatment with Atglistatin. Data represent mean ± SD, Statistical analysis was performed using one-way ANOVA followed by Tukey’s multiple comparisons test to perform intergroup comparisons. The following groups were compared to each other: control vs. KO, control vs. control Atglistatin, control Atglistatin vs. KO Atglistatin, KO vs. KO Atglistatin. Correlation between cardiac neutrophil number and EF was tested by Pearson correlation. Reported are *p* values below 0.05. *p < 0.05, **p < 0.01, ***p < 0.001

Due to the lipid–neutrophil co-localization, we speculated that neutrophil infiltration was a direct or indirect consequence of cardiac lipid accumulation in iCMp38αKO hearts. Assuming that the excess cardiac lipids were derived from adipose tissue lipolysis we used Atglistatin, an inhibitor of adiposetriglyceride lipase (ATGL) [30, 52], the key enzyme regulating triglyceride lipolysis. Baseline cardiac function as well as stimulation of the angiotensin II pathway was not affected by Atglistatin treatment (Supplemental Table S5, Supplemental Fig. S4A). Lipolysis was increased in iCMp38αKO animals after AngII treatment as depicted by elevated plasma glycerol levels (control: 0.074 ± 0.04 mM, KO: 0.22 ± 0.01 mM). Additional Atglistatin treatment attenuated plasma glycerol levels nearly by half in KO animals (control Atglistatin: 0.075 ± 0.02 mM, KO Atglistatin: 0.13 ± 0.01 mM) (Fig. 5B). Inhibition of lipolysis also reduced lipid accumulation in iCMp38αKO hearts (Fig. 5C), proving our hypothesis that cardiac lipids accumulating in the hearts were derived from peripheral lipolysis. Moreover, pretreatment with Atglistatin also improved cardiac function as EF and FS increased by ~ 30% (EF: 26.9 ± 5.1%, FS: 9.9 ± 2.6%) in comparison with vehicle-treated iCMp38αKO hearts (EF: 20.5 ± 4.9%, FS: 7.1 ± 2.3%) (Fig. 5D). In line with an increase in contractile function SV increased, and ESV and EDV were shifted slightly to lower values suggesting a reduced dilation (Supplemental Fig. S4B).

Lipolysis inhibition also modulated the inflammatory response. As shown in Fig. 5E, AngII treatment resulted in a substantial increase in neutrophils (CD11b^+^Ly6G^+^) in the hearts of iCMp38αKO hearts. Similarly, monocyte (CD11b^+^Ly6G^+^CD64^−^MHCII^−^) and macrophage (CD11b^+^Ly6G^+^CD64^+^) numbers were higher in iCMp38αKO hearts after AngII treatment. This increase was mainly due to Ly6C^hi^ monocytes and macrophages. Analysis of CCR2 as a marker for monocyte-derived macrophages showed that the resident CCR2^−^ macrophages were not altered, but monocyte-derived CCR2 + macrophages were almost doubled. Also, B-cells (CD45^+^CD19^+^) as well as CD4^+^- and CD8^+^-T-cells (CD45^+^CD3^+^) were elevated, which underscores the development of a proinflammatory milieu in AngII-treated iCMp38αKO hearts (Fig. 5F). Inhibition of lipolysis by Atglistatin reduced neutrophil numbers by 30% and showed a strong inversely proportional correlation between the number of neutrophils and ejection fraction (Fig. 5G). It also appeared to attenuate monocytes, CCR2 + macrophages, B- and T-cells, although the consistent reduction of their mean values did not reach statistical significance. Thus, lipid accumulation due to loss of p38 MAPKα activity promoted a proinflammatory response to AngII-induced PO, which contributed to systolic failure.

Since sympathetic overstimulation can exacerbate cardiac function, we treated animals with the unspecific β1/2-adrenergic receptor antagonist propranolol for 1 day before AngII pump implantation and during AngII infusion. Cardiac systolic pump function as measured by ejection fraction (KO vehicle: 27 ± 4.6%, KO propranolol: 34.3 ± 8.9%) and fractional shortening (KO vehicle: 10.2 ± 0.9%, KO propranolol: 12.2 ± 2.8%) trended to be improved in propranolol-treated animals compared to vehicle but did not reach statistical significance. In addition, neutrophil accumulation in the hearts of iCMp38αKO mice were still elevated under propranolol treatment, indicating that the increased sympathetic tone is not the primary cause for cardiac dysfunction in iCMp38αKO mice (Supplemental Fig. S5).

### Depletion of neutrophil granulocytes improves cardiac function in p38 MAPKα deficient hearts

**Fig. 6:**
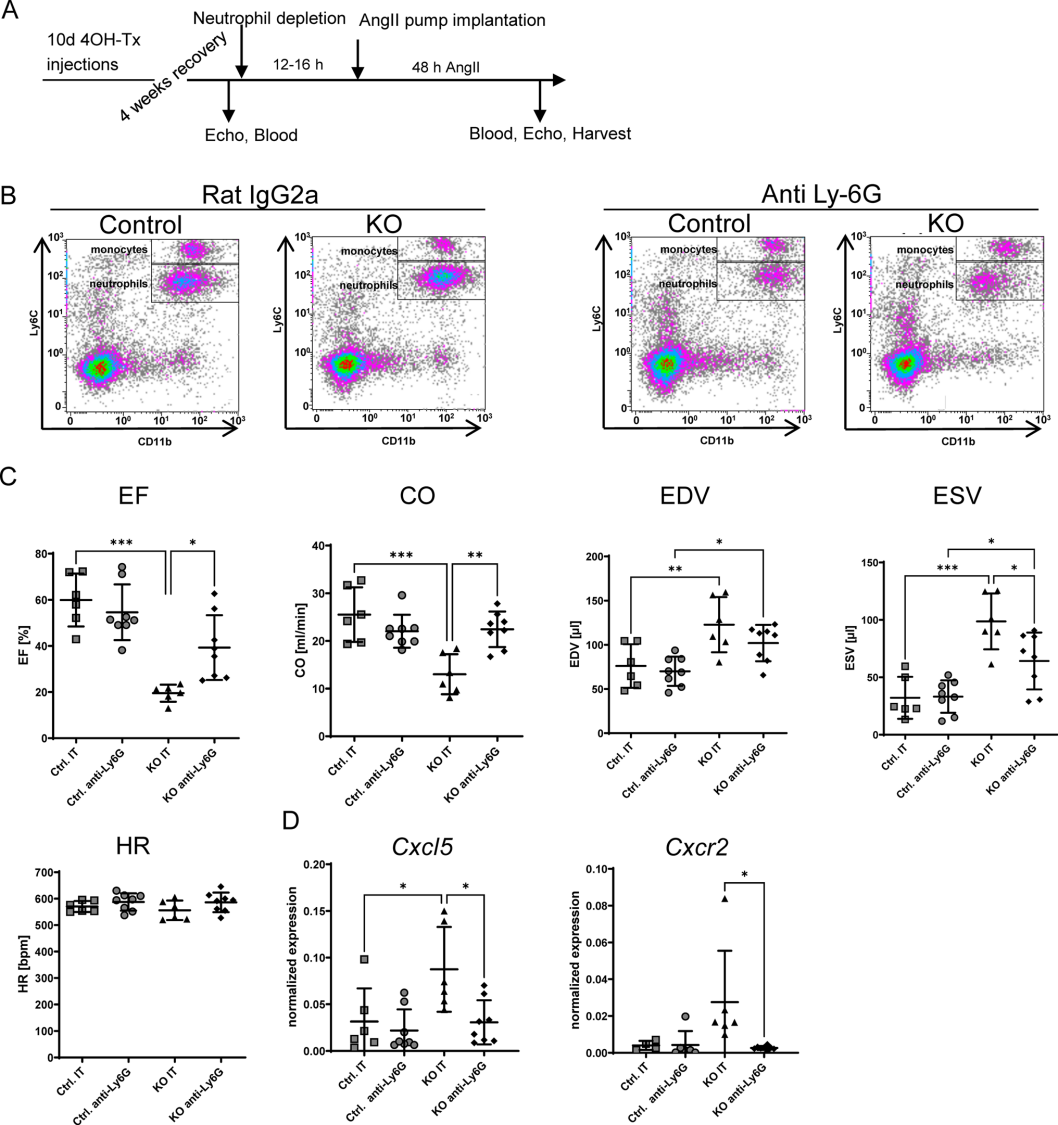
Granulocyte depletion: **A** schematic of experimental outline. **B** FACS analysis of neutrophils in blood of control and iCMp38αKO mice 60–64 h after treatment with anti-Ly6G antibody or isotype control (Rat IgG2A). Representative FACS plots are shown. **C** EF, cardiac output (CO), EDV, ESV and heart rate of control and iCMp38αKO mice after 48 h AngII treatment with either anti-Ly6G antibody or isotype control (IT). *n* = 6–8. **D** Gene expression level of *Cxcl5* and *Cxcr2* in control and iCMp38αKO hearts after 48 h AngII treatment with either anti-Ly6G antibody or isotype control (IT). *n* = 4–8, Statistical analysis was performed using one-way ANOVA followed by Tukey’s multiple comparisons test to perform intergroup comparisons. The following groups were compared to each other: control IT vs. KO IT, control IT vs. control anti-Ly6G, control anti-Ly6G vs. KO anti-Ly6G, KO IT vs. KO anti-Ly6G. Reported are *p* values below 0.05 *p < 0.05, **p < 0.01, ***p < 0.001

To further investigate the impact of neutrophil infiltration on cardiac function and dilation in iCMp38αKO mice, neutrophils were depleted by application of anti-Ly6G antibody (anti-Ly6G) or isotype control (IT). 12–16 h after antibody injection mice received AngII for 48 h (Fig. 6A). FACS analysis of blood samples showed a successful depletion of neutrophils in anti-Ly6G but not IT-treated controls (Fig. 6B).

Analysis of cardiac function revealed that starting at a comparable basal function (Supplemental Table S6), IT-treated iCMp38αKO mice developed the characteristic LV dilation and reduced cardiac output after 48 h AngII, whereas IT-treated controls had a normal cardiac function (Fig. 6C). Neutrophil depletion did not affect cardiac function in AngII-treated control mice; however, it substantially improved systolic pump function in AngII-treated iCMp38αKO mice, since EF increased and cardiac output (CO) was normalized in comparison with IT-treated iCMp38αKO mice (Fig. 6C EF, CO). Neutrophil depletion also normalized the expression of *Cxcl5* and its corresponding receptor *Cxcr2*, which were upregulated in hearts of iCMp38αKO mice after 48 h AngII-treatment (Fig. 6D). Moreover, lipid accumulation was still detected in the majority of hearts analyzed (Supplemental Fig. S6), demonstrating that lipid deposition occurred prior to neutrophil infiltration supporting the hypothesis, that lipid accumulation, at least in part, caused neutrophil infiltration.

## Discussion

Pressure overload (PO) results in a pathologic type of cardiac hypertrophy, involving concentric growth of the left ventricle [58] and cardiac fibrosis [13], which may eventually promote the development of heart failure. The protein kinase p38 MAPKα belongs to a family of stress kinases which is rapidly activated in response to elevated mechanical load in cardiac myocytes. Therefore, its role in PO hypertrophy has been addressed in a variety of mouse models including the overexpression of dominant negative forms of the p38 upstream regulators [4] and cardiomyocyte specific knockouts [37, 46]. Depending on the model, downregulation of p38 MAPKα activity was inferred to promote a hypertrophic response via NFAT-calcineurin signalling [4], or to cause LV dilatation due to a higher susceptibility of myocytes to cell death [37]. Thus, no clear picture emerged which defines the role of p38 MAPKα in PO hypertrophy.

In this report we provide evidence that loss of CM p38 MAPKα expression results in a functional and metabolic depression of the heart in response to PO enhancing adipose tissue lipolysis, lipid deposition in cardiac myocytes, and myocardial neutrophil infiltration and inflammation as early as 2 days after onset of AngII application. Inhibition of adipose tissue lipolysis reduced lipid droplet accumulation, attenuated immune cell infiltration, and improved cardiac function providing a link between cardiac metabolic dysfunction and sterile inflammation. Moreover, depletion of neutrophils, the major immune cell type infiltrating the p38α-deficient heart, resulted in an even more pronounced rescue of heart function demonstrating that invading neutrophils were a major cause of heart failure development. Thus, the mechanisms leading to heart failure in cardiomyocyte-specific p38 MAPKα KO mice are not restricted to the cardiomyocytes themselves but also involve a systemic crosstalk between cardiomyocytes, adipose tissue, and the immune system.

The observed AngII-induced rapid LV dilation of p38 MAPKα KO hearts was associated with fundamental alterations of the cardiac gene expression pattern. Hearts with induced p38 MAPKα deficiency revealed a set of > 3400 cardiac transcripts which were differentially expressed after AngII treatment. Among these, a large number of genes involved in cardiac metabolism, as, for example, TCA cycle, fatty acid synthesis or respiratory chain function were dysregulated in p38 MAPKα KO hearts. IPA analysis identified several upstream regulators which might orchestrate the observed transcriptomic changes: *Ppargc1a* (Pgc1α), *Ppargc1b* (Pgc1β), *Pparα*, and *Pparγ* belonged to a group of transcriptional activators with negative activation Z-score, and we confirmed a reduced expression of the master regulator of mitochondrial biogenesis, Pgc1α, in p38α-deficient hearts. Among the upstream regulators with a positive *Z*-score, the tumor suppressor p53 was identified. Recently, cardiac specific p53 KO mice revealed an increased expression of a metabolic gene expression program, including a direct regulation of *Ppargc1a* [28], and, therefore, shows in part an inverse phenotype of p38α KO hearts. In terms of gene expression, p38α KO hearts further show an intriguing homology to human arrhythmogenic right ventricular cardiomyopathy (ARVC) and dilative cardiomyopathy (DCM). In a combined proteomic/transcriptomic analysis Chen et al. [9] found a common set of upstream regulators shared by both pathologies. Of note, the upstream regulators *Rictor, Kdm5a, Mapkap4, Tgfb3, Tgfb1, Ppargc1a, Insr, Mtpn* which were discovered in the AVRC/DCM study were also among the top regulators identified in the dilated iCMp38αKO hearts with an absolute *Z*-score > 2 and the same direction of activity. Thus, the common upstream regulators found in the human ARVC/DCM hearts and in the AngII-iCMp38αKO model may represent a general signature for the development of heart failure. In contrast to the extensively altered gene expression profile after 48 h AngII induction, transcriptomic data obtained after 12 h of AngII treatment revealed only minor changes. Notably, “oxidative phosphorylation” was also affected at this early timepoint, albeit to a lower extent. However, this may indicate that depressed mitochondrial function contributes to the progression to cardiac dilation occurring at 48 h.

In line with the gene expression data, the deposition of perilipin-coated lipid droplets in cardiac myocytes and an elevated content of fatty acids in p38 MAPKα KO hearts point to a critical role of p38 MAPKα in cardiac metabolic adaptation to stress. Lipid droplets trap surplus fatty acids as triacylglycerol (TAG) under conditions of elevated fatty acid synthesis, enhanced lipid uptake, or reduced β-oxidation. The lipid storage as TAG in lipid droplets reduces cytoplasmic fatty acids which otherwise may be metabolized to sphingosines and ceramides, which alter signalling functions or even induce lipotoxicity [14, 22]. The lipid droplet accumulation in p38 MAPKα KO hearts could result from a reduced oxidative capacity because of a concerted down regulation of mitochondrial genes. On the other hand, an increased fatty acid supply could promote fatty acid accumulation in p38 MAPKα KO hearts. AngII increases systemic vascular resistance, which requires a higher cardiac work to overcome the elevated vasoconstriction. Usually, this response is mediated by an increased sympathetic tone to enhance cardiac contractility. However, β-adrenergic stimulation also induces lipolysis in peripheral adipose tissue [64], a response also known as stress-induced lipolysis [44]. Serum glycerol levels, as a measure for adipose tissue lipolysis, were elevated in iCMp38αKO animals, and this response was attenuated by inhibiting adipose tissue lipolysis using the small molecule inhibitor of ATGL, Atglistatin, which blocks this enzyme preferentially in adipocytes [30, 52]. Concomitantly, lipid accumulation in cardiac myocytes was almost fully suppressed. Thus, lipid accumulation in AngII-treated iCMp38αKO hearts critically depended on adipose tissue lipolysis.

Our kinetic analysis revealed that lipids accumulated in control hearts at 12 h after onset of AngII treatment, indicating that an early imbalance of lipid uptake and oxidation occurred independent of p38 MAPKα inactivation and coincided with a transient decline in contractile function. Lipid droplets vanished after 24 h when cardiac function recovered. In contrast, iCMp38αKO hearts were unable to adapt functionally and developed a dilation of the left ventricle which was already detectable at 24 h. Of note, lipid deposition increased from the onset of AngII treatment and persisted on high levels at later timepoints.

Another novel finding is the extensive involvement of neutrophils to the PO induced modulation of iCMp38αKO hearts. Inactivation of p38 MAPKα in cardiac myocytes induces a remarkable shift in the immune cell infiltration and, in particular, neutrophils are highly elevated on day 2 after onset of PO in hearts of iCMp38αKO mice. Also, the numbers of pro-inflammatory Ly6C^hi^ monocytes and macrophages as well as B- and T-cells were higher on day 2 of AngII treatment. Thus, the loss of p38 MAPKα in cardiac myocytes resulted in a pro-inflammatory environment with a pronounced contribution of neutrophils. PO caused by TAC and AngII, respectively, is associated with immune cell infiltration involving mainly CCR2^+^ monocyte derived macrophages [8, 42, 62], T- and B-cells [5]. All of these cell types contribute to ventricular dysfunction, cardiomyocyte hypertrophy and the progression from hypertrophy to failure. Neutrophils are usually major players of cardiac inflammation in ischemic injury, and were generally thought to play a minor role in PO hypertrophy, as also seen here in control hearts. However, in the absence of CM p38α expression these cells take over a predominant role in the development of LV dilation.

A striking finding in the iCMp38αKO model is the tight spatial relationship of lipid deposition in CM and neutrophil infiltration. Apparently, the failure of metabolic and functional adaptation by p38α deficient hearts induces the release of chemotactic signals, which attract granulocytes to the sites of “injury” including IL1β, HMGB1 and others. The chemical identity of the chemoattractant was not identified here but we propose that metabolic depression led to the release of molecules acting as damage-associated molecular patterns, which attract granulocytes. Moreover, earlier work has shown that β-adrenergic stimulation affects leukocyte composition and function in HF [27]. However, in our experiments using the β1/2-adrenergic antagonist propranolol in combination with AngII, at least cardiac neutrophil infiltration was similar with and without propranolol, suggesting that other mechanisms were responsible for neutrophil attraction. Besides affecting cardiac function, propranolol might also inhibit white adipose tissue lipolysis. However, lipolysis in rodents depends to a large extent on β3-adrenergic receptor activation, limiting the potential of the β1/2 adrenoceptor antagonist propranolol and other generally used β-adrenoceptor antagonists to study the relationship of β-adrenergic stimulation, lipolysis, and cardiac lipid accumulation. Moreover, natriuretic peptides, released under HF conditions, also potently elevate lipolysis [7], which might further complicate the deduction of a causal relationship of SNS activation and lipolysis in iCMp38αKO mice.

Fatty acids released from damaged cardiac myocytes may represent a possible source of chemoattractants, because triacylglycerol and long chain fatty acids have chemoattractant function via TLR4 mediated signalling [11]. In line with this, iCMp38αKO hearts also showed a higher number of apoptotic cells. The pro-inflammatory role of lipid deposition was corroborated by inhibition of lipolysis using Atglistatin. This intervention not only attenuated cardiac lipid accumulation but also reduced neutrophils by > 30%. Monocytes, macrophages, and T- and B-cells were almost brought to control values, and cardiac function was improved. Therefore, ectopic lipid deposition in the heart due to stimulated adipose tissue lipolysis and concomitant metabolic dysfunction represents an important stimulus for the attraction of immune cells. In addition, cytokines and chemokines, which were upregulated in the hearts of AngII-treated iCMp38αKO animals, could attract immune cells.

The impact of adipose tissue lipolysis and therefore, a heart–adipose crosstalk in cardiac remodelling was also demonstrated in adipose tissue specific ATGL KO mice [41, 47] subject to TAC. When ATGL was inactivated an improvement of cardiac function, less hypertrophy and fibrosis were found. Importantly, cardiac lipid profiles, which were substantially altered in WT hearts after TAC were similar to control conditions in adipose ATGL KO mice. Unfortunately, it was not reported to what extent the immune cell content in these hearts was modulated.

Because ATGL inhibition improved cardiac function, attenuated cardiac lipid deposition and granulocyte infiltration the question arose whether lipids and/or neutrophils caused the functional depression in iCMp38αKO hearts. Depletion of granulocytes shortly before AngII treatment abrogated inflammation and contractile dysfunction more efficient than inhibition of lipolysis demonstrating that neutrophils were the major cause of cardiac dilation and contractile dysfunction. As lipid droplet formation was still observed, granulocyte infiltration was rather secondary to metabolic dysfunction than an inducer of lipid deposition. It is important to note that also myocardial infarction is accompanied by an early lipid droplet accumulation as in our iCMp38αKO animals after AngII treatment. Indeed, we could show that blockade of adipocyte lipolysis improved the outcome post MI [3]. This opens the interesting option that the extensive neutrophil invasion occurring after ischemia may be triggered in part by elevated lipolysis. Therefore, modulation of adipose tissue lipolysis may represent a novel therapeutic principle to affect the sterile inflammation in various cardiac pathological conditions. The findings presented in this paper reveal novel mechanisms by which p38 MAPKα expressed in cardiac myocytes, plays a protective role very early after the onset of increased afterload. Most studies trying to elucidate the function of p38 MAPKα focussed on the analysis of late events such as cardiomyocyte hypertrophy and fibrosis. In that, the important early metabolic and inflammatory triggers of the late outcome were not addressed. Thus, loss of CM p38α resulted in an altered crosstalk of cardiac myocytes with the immune system and adipose tissue which substantially affected the cardiac phenotype. In line with this finding, Rose et al. recently found that cardiomyocyte p38 MAPKα regulates a CM–endothelial cell crosstalk which leads to an enhanced angiogenesis in response to pressure overload by the release of VEGF [46]. Thus, the inactivation of p38 MAPKα in cardiac myocytes represents an interesting model to investigate intercellular crosstalk in the context of cardiac hypertrophy and heart failure.

In terms of translational impact, our results indicate to act with caution when introducing p38 MAPK inhibitors into the clinics. We show that p38 MAPKα plays a protective role in cardiac myocytes and inhibition of its activity may negatively affect cardiac function. On the other hand, fibroblast-specific inactivation of p38 MAPKα attenuated the formation of cardiac myofibroblasts and the development of interstitial fibrosis in PO induced hypertrophy indicating a possible therapeutic impact of p38 MAPK inhibition in the treatment of cardiac fibrosis [35]. Interestingly, earlier phase III clinical trials failed to show an improvement in the outcome after myocardial infarction by losmapimod [31, 38]. However, p38 MAPK inhibitors, as losmapimod, are investigated as promising anti-inflammatory drugs for treatment of facioscapulohumeral muscular dystrophy (NCT04003974) or COVID-19 (NCT04511819). Taken together with the here presented data, the use of p38 MAPK inhibitors as therapeutic agents should be thoroughly investigated for cardiac side effects.

## Supplementary Information

Below is the link to the electronic supplementary material.

Supplementary file1 (PDF 21370 kb)

## Data Availability

Raw microarray data are available via the GEO repository GSE86074.

## References

[1] Bacmeister L, Schwarzl M, Warnke S, Stoffers B, Blankenberg S, Westermann D, Lindner D (2019). Inflammation and fibrosis in murine models of heart failure. Basic Res Cardiol.

[2] Bolstad BM, Irizarry RA, Astrand M, Speed TP (2003). A comparison of normalization methods for high density oligonucleotide array data based on variance and bias. Bioinformatics.

[3] Bottermann K, Granade ME, Oenarto V, Fischer JW, Harris TE (2020). Atglistatin pretreatment preserves remote myocardium function following myocardial infarction. J Cardiovasc Pharmacol Ther.

[4] Braz JC, Bueno OF, Liang Q, Wilkins BJ, Dai YS, Parsons S, Braunwart J, Glascock BJ, Klevitsky R, Kimball TF, Hewett TE, Molkentin JD (2003). Targeted inhibition of p38 MAPK promotes hypertrophic cardiomyopathy through upregulation of calcineurin-NFAT signaling. J Clin Invest.

[5] Brenes-Castro D, Castillo EC, Vazquez-Garza E, Torre-Amione G, Garcia-Rivas G (2018). Temporal frame of immune cell infiltration during heart failure establishment: lessons from animal models. Int J Mol Sci.

[6] Breslin WL, Strohacker K, Carpenter KC, Haviland DL, McFarlin BK (2013). Mouse blood monocytes: standardizing their identification and analysis using CD115. J Immunol Methods.

[7] Ceddia RP, Collins S (2020). A compendium of G-protein-coupled receptors and cyclic nucleotide regulation of adipose tissue metabolism and energy expenditure. Clin Sci (Lond).

[8] Chen B, Frangogiannis NG (2016). Macrophages in the remodeling failing heart. Circ Res.

[9] Chen L, Yang F, Chen X, Rao M, Zhang NN, Chen K, Deng H, Song JP, Hu SS (2017). Comprehensive myocardial proteogenomics profiling reveals C/EBPalpha as the key factor in the lipid storage of ARVC. J Proteome Res.

[10] Cordero-Reyes AM, Youker KA, Trevino AR, Celis R, Hamilton DJ, Flores-Arredondo JH, Orrego CM, Bhimaraj A, Estep JD, Torre-Amione G (2016). Full expression of cardiomyopathy is partly dependent on B-cells: a pathway that involves cytokine activation, immunoglobulin deposition, and activation of apoptosis. J Am Heart Assoc.

[11] Eguchi K, Manabe I, Oishi-Tanaka Y, Ohsugi M, Kono N, Ogata F, Yagi N, Ohto U, Kimoto M, Miyake K, Tobe K, Arai H, Kadowaki T, Nagai R (2012). Saturated fatty acid and TLR signaling link beta cell dysfunction and islet inflammation. Cell Metab.

[12] Fischer TA, Ludwig S, Flory E, Gambaryan S, Singh K, Finn P, Pfeffer MA, Kelly RA, Pfeffer JM (2001). Activation of cardiac c-Jun NH(2)-terminal kinases and p38-mitogen-activated protein kinases with abrupt changes in hemodynamic load. Hypertension.

[13] Frangogiannis NG (2019). Cardiac fibrosis: cell biological mechanisms, molecular pathways and therapeutic opportunities. Mol Aspects Med.

[14] Goldberg IJ, Trent CM, Schulze PC (2012). Lipid metabolism and toxicity in the heart. Cell Metab.

[15] Han J, Molkentin JD (2000). Regulation of MEF2 by p38 MAPK and its implication in cardiomyocyte biology. Trends Cardiovasc Med.

[16] Hayashida W, Kihara Y, Yasaka A, Inagaki K, Iwanaga Y, Sasayama S (2001). Stage-specific differential activation of mitogen-activated protein kinases in hypertrophied and failing rat hearts. J Mol Cell Cardiol.

[17] Heinen A, Godecke S, Flogel U, Miklos D, Bottermann K, Spychala A, Godecke A (2021). 4-hydroxytamoxifen does not deteriorate cardiac function in cardiomyocyte-specific MerCreMer transgenic mice. Basic Res Cardiol.

[18] Heinen A, Nederlof R, Panjwani P, Spychala A, Tschaidse T, Reffelt H, Boy J, Raupach A, Godecke S, Petzsch P, Kohrer K, Grandoch M, Petz A, Fischer JW, Alter C, Vasilevska J, Lang P, Godecke A (2019). IGF1 treatment improves cardiac remodeling after infarction by targeting myeloid cells. Mol Ther.

[19] Heinen A, Raupach A, Behmenburg F, Holscher N, Flogel U, Kelm M, Kaisers W, Nederlof R, Huhn R, Godecke A (2018). Echocardiographic analysis of cardiac function after infarction in mice: validation of single-plane long-axis view measurements and the bi-plane Simpson method. Ultrasound Med Biol.

[20] Heusch P, Canton M, Aker S, van de Sand A, Konietzka I, Rassaf T, Menazza S, Brodde OE, Di Lisa F, Heusch G, Schulz R (2010). The contribution of reactive oxygen species and p38 mitogen-activated protein kinase to myofilament oxidation and progression of heart failure in rabbits. Br J Pharmacol.

[21] Jaeger BN, Donadieu J, Cognet C, Bernat C, Ordonez-Rueda D, Barlogis V, Mahlaoui N, Fenis A, Narni-Mancinelli E, Beaupain B, Bellanne-Chantelot C, Bajenoff M, Malissen B, Malissen M, Vivier E, Ugolini S (2012). Neutrophil depletion impairs natural killer cell maturation, function, and homeostasis. J Exp Med.

[22] Jelenik T, Flogel U, Alvarez-Hernandez E, Scheiber D, Zweck E, Ding Z, Rothe M, Mastrototaro L, Kohlhaas V, Kotzka J, Knebel B, Muller-Wieland D, Moellendorf S, Godecke A, Kelm M, Westenfeld R, Roden M, Szendroedi J (2018). Insulin resistance and vulnerability to cardiac ischemia. Diabetes.

[23] Kaiser RA, Bueno OF, Lips DJ, Doevendans PA, Jones F, Kimball TF, Molkentin JD (2004). Targeted inhibition of p38 mitogen-activated protein kinase antagonizes cardiac injury and cell death following ischemia-reperfusion in vivo. J Biol Chem.

[24] Liang Q, Molkentin JD (2003). Redefining the roles of p38 and JNK signaling in cardiac hypertrophy: dichotomy between cultured myocytes and animal models. J Mol Cell Cardiol.

[25] Livak KJ, Schmittgen TD (2001). Analysis of relative gene expression data using real-time quantitative PCR and the 2(−Delta Delta C(T)) method. Methods.

[26] Maik-Rachline G, Lifshits L, Seger R (2020). Nuclear P38: roles in physiological and pathological processes and regulation of nuclear translocation. Int J Mol Sci.

[27] Maisel AS, Knowlton KU, Fowler P, Rearden A, Ziegler MG, Motulsky HJ, Insel PA, Michel MC (1990). Adrenergic control of circulating lymphocyte subpopulations. effects of congestive heart failure, dynamic exercise, and terbutaline treatment. J Clin Invest.

[28] Mak TW, Hauck L, Grothe D, Billia F (2017). p53 regulates the cardiac transcriptome. Proc Natl Acad Sci USA.

[29] Matyash V, Liebisch G, Kurzchalia TV, Shevchenko A, Schwudke D (2008). Lipid extraction by methyl-tert-butyl ether for high-throughput lipidomics. J Lipid Res.

[30] Mayer N, Schweiger M, Romauch M, Grabner GF, Eichmann TO, Fuchs E, Ivkovic J, Heier C, Mrak I, Lass A, Hofler G, Fledelius C, Zechner R, Zimmermann R, Breinbauer R (2013). Development of small-molecule inhibitors targeting adipose triglyceride lipase. Nat Chem Biol.

[31] Melloni C, Sprecher DL, Sarov-Blat L, Patel MR, Heitner JF, Hamm CW, Aylward P, Tanguay JF, DeWinter RJ, Marber MS, Lerman A, Hasselblad V, Granger CB, Newby LK (2012). The study of LoSmapimod treatment on inflammation and InfarCtSizE (SOLSTICE): design and rationale. Am Heart J.

[32] Michel MC, Li Y, Heusch G (2001). Mitogen-activated protein kinases in the heart. Naunyn Schmiedebergs Arch Pharmacol.

[33] Mizote I, Yamaguchi O, Hikoso S, Takeda T, Taneike M, Oka T, Tamai T, Oyabu J, Matsumura Y, Nishida K, Komuro I, Hori M, Otsu K (2010). Activation of MTK1/MEKK4 induces cardiomyocyte death and heart failure. J Mol Cell Cardiol.

[34] Molina CE, Jacquet E, Ponien P, Munoz-Guijosa C, Baczko I, Maier LS, Donzeau-Gouge P, Dobrev D, Fischmeister R, Garnier A (2018). Identification of optimal reference genes for transcriptomic analyses in normal and diseased human heart. Cardiovasc Res.

[35] Molkentin JD, Bugg D, Ghearing N, Dorn LE, Kim P, Sargent MA, Gunaje J, Otsu K, Davis J (2017). Fibroblast-specific genetic manipulation of p38 mitogen-activated protein kinase in vivo reveals its central regulatory role in fibrosis. Circulation.

[36] Naga Prasad SV, Esposito G, Mao L, Koch WJ, Rockman HA (2000). Gbetagamma-dependent phosphoinositide 3-kinase activation in hearts with in vivo pressure overload hypertrophy. J Biol Chem.

[37] Nishida K, Yamaguchi O, Hirotani S, Hikoso S, Higuchi Y, Watanabe T, Takeda T, Osuka S, Morita T, Kondoh G, Uno Y, Kashiwase K, Taniike M, Nakai A, Matsumura Y, Miyazaki J, Sudo T, Hongo K, Kusakari Y, Kurihara S, Chien KR, Takeda J, Hori M, Otsu K (2004). p38alpha mitogen-activated protein kinase plays a critical role in cardiomyocyte survival but not in cardiac hypertrophic growth in response to pressure overload. Mol Cell Biol.

[38] O'Donoghue ML, Glaser R, Cavender MA, Aylward PE, Bonaca MP, Budaj A, Davies RY, Dellborg M, Fox KA, Gutierrez JA, Hamm C, Kiss RG, Kovar F, Kuder JF, Im KA, Lepore JJ, Lopez-Sendon JL, Ophuis TO, Parkhomenko A, Shannon JB, Spinar J, Tanguay JF, Ruda M, Steg PG, Theroux P, Wiviott SD, Laws I, Sabatine MS, Morrow DA, Investigators L-T (2016). Effect of losmapimod on cardiovascular outcomes in patients hospitalized with acute myocardial infarction: a randomized clinical trial. JAMA.

[39] Otsu K, Yamashita N, Nishida K, Hirotani S, Yamaguchi O, Watanabe T, Hikoso S, Higuchi Y, Matsumura Y, Maruyama M, Sudo T, Osada H, Hori M (2003). Disruption of a single copy of the p38alpha MAP kinase gene leads to cardioprotection against ischemia-reperfusion. Biochem Biophys Res Commun.

[40] Ozbalci C, Sachsenheimer T, Brugger B (2013). Quantitative analysis of cellular lipids by nano-electrospray ionization mass spectrometry. Methods Mol Biol.

[41] Parajuli N, Takahara S, Matsumura N, Kim TT, Ferdaoussi M, Migglautsch AK, Zechner R, Breinbauer R, Kershaw EE, Dyck JRB (2018). Atglistatin ameliorates functional decline in heart failure via adipocyte-specific inhibition of adipose triglyceride lipase. Am J Physiol Heart Circ Physiol.

[42] Patel B, Bansal SS, Ismahil MA, Hamid T, Rokosh G, Mack M, Prabhu SD (2018). CCR2(+) monocyte-derived infiltrating macrophages are required for adverse cardiac remodeling during pressure overload. JACC Basic Transl Sci.

[43] Pinto AR, Ilinykh A, Ivey MJ, Kuwabara JT, D'Antoni ML, Debuque R, Chandran A, Wang L, Arora K, Rosenthal NA, Tallquist MD (2016). Revisiting cardiac cellular composition. Circ Res.

[44] Raje V, Ahern KW, Martinez BA, Howell NL, Oenarto V, Granade ME, Kim JW, Tundup S, Bottermann K, Godecke A, Keller SR, Kadl A, Bland ML, Harris TE (2020). Adipocyte lipolysis drives acute stress-induced insulin resistance. Sci Rep.

[45] Romero-Becerra R, Santamans AM, Folgueira C, Sabio G (2020). p38 MAPK pathway in the heart: new insights in health and disease. Int J Mol Sci.

[46] Rose BA, Yokota T, Chintalgattu V, Ren S, Iruela-Arispe L, Khakoo AY, Minamisawa S, Wang Y (2017). Cardiac myocyte p38alpha kinase regulates angiogenesis via myocyte-endothelial cell cross-talk during stress-induced remodeling in the heart. J Biol Chem.

[47] Salatzki J, Foryst-Ludwig A, Bentele K, Blumrich A, Smeir E, Ban Z, Brix S, Grune J, Beyhoff N, Klopfleisch R, Dunst S, Surma MA, Klose C, Rothe M, Heinzel FR, Krannich A, Kershaw EE, Beule D, Schulze PC, Marx N, Kintscher U (2018). Adipose tissue ATGL modifies the cardiac lipidome in pressure-overload-induced left ventricular failure. PLoS Genet.

[48] Sasse A, Wallich M, Ding Z, Goedecke A, Schrader J (2003). Coxsackie-and-adenovirus receptor mRNA expression in human heart failure. J Gene Med.

[49] Schulz R, Aker S, Belosjorow S, Konietzka I, Rauen U, Heusch G (2003). Stress kinase phosphorylation is increased in pacing-induced heart failure in rabbits. Am J Physiol Heart Circ Physiol.

[50] Schulz R, Belosjorow S, Gres P, Jansen J, Michel MC, Heusch G (2002). p38 MAP kinase is a mediator of ischemic preconditioning in pigs. Cardiovasc Res.

[51] Schulz R, Gres P, Skyschally A, Duschin A, Belosjorow S, Konietzka I, Heusch G (2003). Ischemic preconditioning preserves connexin 43 phosphorylation during sustained ischemia in pig hearts in vivo. FASEB J.

[52] Schweiger M, Romauch M, Schreiber R, Grabner GF, Hutter S, Kotzbeck P, Benedikt P, Eichmann TO, Yamada S, Knittelfelder O, Diwoky C, Doler C, Mayer N, De Cecco W, Breinbauer R, Zimmermann R, Zechner R (2017). Pharmacological inhibition of adipose triglyceride lipase corrects high-fat diet-induced insulin resistance and hepatosteatosis in mice. Nat Commun.

[53] Sohal DS, Nghiem M, Crackower MA, Witt SA, Kimball TR, Tymitz KM, Penninger JM, Molkentin JD (2001). Temporally regulated and tissue-specific gene manipulations in the adult and embryonic heart using a tamoxifen-inducible Cre protein. Circ Res.

[54] Subkhankulova T, Mitchell SA, Willis AE (2001). Internal ribosome entry segment-mediated initiation of c-Myc protein synthesis following genotoxic stress. Biochem J.

[55] Takeishi Y, Huang Q, Abe J, Glassman M, Che W, Lee JD, Kawakatsu H, Lawrence EG, Hoit BD, Berk BC, Walsh RA (2001). Src and multiple MAP kinase activation in cardiac hypertrophy and congestive heart failure under chronic pressure-overload: comparison with acute mechanical stretch. J Mol Cell Cardiol.

[56] Tenhunen O, Sarman B, Kerkela R, Szokodi I, Papp L, Toth M, Ruskoaho H (2004). Mitogen-activated protein kinases p38 and ERK 1/2 mediate the wall stress-induced activation of GATA-4 binding in adult heart. J Biol Chem.

[57] Thomsen R, Solvsten CA, Linnet TE, Blechingberg J, Nielsen AL (2010). Analysis of qPCR data by converting exponentially related *C*_t_ values into linearly related X0 values. J Bioinform Comput Biol.

[58] van Berlo JH, Maillet M, Molkentin JD (2013). Signaling effectors underlying pathologic growth and remodeling of the heart. J Clin Invest.

[59] Ventura JJ, Tenbaum S, Perdiguero E, Huth M, Guerra C, Barbacid M, Pasparakis M, Nebreda AR (2007). p38alpha MAP kinase is essential in lung stem and progenitor cell proliferation and differentiation. Nat Genet.

[60] Verrou C, Zhang Y, Zurn C, Schamel WW, Reth M (1999). Comparison of the tamoxifen regulated chimeric Cre recombinases MerCreMer and CreMer. Biol Chem.

[61] Wang Y, Sano S, Oshima K, Sano M, Watanabe Y, Katanasaka Y, Yura Y, Jung C, Anzai A, Swirski FK, Gokce N, Walsh K (2019). Wnt5a-mediated neutrophil recruitment has an obligatory role in pressure overload-induced cardiac dysfunction. Circulation.

[62] Xia Y, Lee K, Li N, Corbett D, Mendoza L, Frangogiannis NG (2009). Characterization of the inflammatory and fibrotic response in a mouse model of cardiac pressure overload. Histochem Cell Biol.

[63] Yokota T, Wang Y (2016). p38 MAP kinases in the heart. Gene.

[64] Young SG, Zechner R (2013). Biochemistry and pathophysiology of intravascular and intracellular lipolysis. Genes Dev.

[65] Zhang S, Ren J, Zhang CE, Treskov I, Wang Y, Muslin AJ (2003). Role of 14-3-3-mediated p38 mitogen-activated protein kinase inhibition in cardiac myocyte survival. Circ Res.

[66] Zhang S, Weinheimer C, Courtois M, Kovacs A, Zhang CE, Cheng AM, Wang Y, Muslin AJ (2003). The role of the Grb2-p38 MAPK signaling pathway in cardiac hypertrophy and fibrosis. J Clin Invest.

