# Supplementary material for: Cardiomyocyte p38 MAPKα suppresses a heart–adipose tissue–neutrophil crosstalk in heart failure development
Source: Basic Res Cardiol. 2022 Oct 7;117(1):48. doi: 10.1007/s00395-022-00955-2 (PMC9542472; doi:10.1007/s00395-022-00955-2)
Supplement: Supplementary file 1 — Supplementary file1 (PDF 21370 kb) [file 395_2022_955_MOESM1_ESM.pdf]

Supplementary Material

Cardiomyocyte p38 MAPKα suppresses a heart – adipose tissue – neutrophil crosstalk in heart failure

Katharina Bottermann[1,4], Lisa Kalfhues[1], Rianne Nederlof[1], Anne Hemmers[1], Lucia M. Leitner[1], Vici Oenarto[1], Jana Nemmer[1], Mirjam Pfeffer[1], Vidisha Raje[2], Rene Deenen[3], Patrick Petzsch[3], Heba Zabri[4], Karl Köhrer[3], Andreas S. Reichert[5], Maria Grandoch[6], Jens W. Fischer[4,9], Diran Herebian[7], Johannes Stegbauer[8], Thurl E. Harris[2], and Axel Gödecke[1,9]

[1]Department of Cardiovascular Physiology, Medical Faculty and University Hospital Düsseldorf, Heinrich-Heine-University Düsseldorf, Postfach 101007, 40001 Düsseldorf, Germany

[2]Department of Pharmacology, University of Virginia Health System, Charlottesville, VA, 22908, USA

[3]Biological and Medical Research Center (BMFZ), Medical Faculty, Heinrich-Heine-University, Universitätsstraße 1, 40225 Duesseldorf, Germany

[4]Institute of Pharmacology, Medical Faculty and University Hospital Düsseldorf, Heinrich-Heine-University Düsseldorf, Postfach 101007, 40001 Düsseldorf, Germany

[5]Institute of Biochemistry and Molecular Biology I, Medical Faculty and University Hospital Düsseldorf, Heinrich-Heine-University Düsseldorf, Postfach 101007, 40001 Düsseldorf, Germany

[6]Institute of Translational Pharmacology, Medical Faculty and University Hospital Düsseldorf, Heinrich-Heine-University Düsseldorf, Postfach 101007, 40001 Düsseldorf, Germany

[7]Department of General Pediatrics, Neonatology and Pediatric Cardiology, Medical Faculty and University Hospital Düsseldorf, Heinrich-Heine-University Düsseldorf, Postfach 101007, 40001 Düsseldorf, Germany

[8]Department of Nephrology, Medical Faculty and University Hospital Düsseldorf, Heinrich-Heine-University Düsseldorf, Postfach 101007, 40001 Düsseldorf, Germany

[9]CARID – Cardiovascular Research Institute Düsseldorf, Medical Faculty, Heinrich-Heine-University, Universitätsstraße 1, 40225 Duesseldorf, Germany

Address for correspondence: Axel Gödecke, Department of Cardiovascular Physiology, Heinrich-Heine-University, University Hospital Düsseldorf, Universitätstraße 1, 40225 Düsseldorf, Postfach 101007, 40001 Düsseldorf, Germany, Phone: +49-211-8112670, Fax: +49-211-8112672, Axel.Goedecke@uni-duesseldorf.de

Supplemental Table S1: Primary and secondary antibodies used in western blot analysis

	Antibodies	Company	No	Dilution
1st	Mouse anti-p38 MAPKα	Cell Signaling	#9212	1:1000
	p38MAPK γ	Cell Signaling	#2307	1:1000
	Rabbit anti-p38 MAPKαT180/Y182	Cell Signaling	#9216	1:1000
	Rabbit anti-MK2T334	Cell Signaling	#3007	1:1000
2nd	IRDye 680RD Goat anti-Mouse IgG	Li-COR	925-68070	1:10 000
	IRDye 800CW Goat anti-Rabbit IgG	Li-COR	925-32211	1:10 000
	IRDye 680RD Goat anti-Rabbit IgG	Li-COR	926-68071	1:10 000
	IRDye 800CW Goat anti-Mouse IgG	Li-COR	926-32210	1:10 000

Supplemental Table S2: Primer sequences

Gene	Primer Orientation	Sequence
Angptl4	Fwd	ATGGAGTAGACAAGACTTCG
	Rev	TCACAGTTGACCAAAAATGG
Cxcl5	Fwd	TGTTTGCTTAACCGTAACTC
	Rev	CAGTTTAGCTATGACTTCCAC
Cxcr2	Fwd	CTACTGCAGGATTAAGTTTACC
	Rev	GACGTATATTACAACCACAGC
Il1b	Fwd	GGATGATGATAACCTGC
	Rev	CATGGAGAATATCACTTGTTGG
Il6	Fwd	AAGAAATGATGGATGCTACC
	Rev	GAGTTTCTGTATCTCTCTGAAG
Nudc	Fwd	AGAACTCCAAGCTATCAGAC
	Rev	CTTCAGGATTTCCTGTTTCTTC
p38 MAPKalpha	Fwd	TCATTCACGCCAAAAGGACC
	Rev	CTGGCACTTCACGATGTTGT
p38 MAPKbeta	Fwd	GACCTGAATAACATCGTCAAG
	Rev	GAGTGGATATACTTCAGCCC
p38 MAPKgamma	Fwd	TGAAGGGGCTGAAGTATATC
	Rev	CATCCAATTCAAGATGACCTC
p38 MAPKdelta	Fwd	CAGAAATGCTGACTGGAAAG
	Rev	CTGAATATAGGATTTGGCCG
Ppargc1a	Fwd	TCCTCTTCAAGATCCTGTTAC
	Rev	CACATACAAGGGAGAATTGC
Pdk4	Fwd	ACAATCAAGATTTCTGACCG
	Rev	TCTCCTTGAAAATACTTGGC
Slc2a4	Fwd	CAATGGTTGGGAAGGAAAAG
	Rev	AATGAGTATCTCAGGAGGC
Tgfb2	Fwd	GAGATTTGCAGGTATTGATGG
	Rev	CAACAACATTAGCAGGAGATG

Supplemental Table S3: Antibodies used for FACS analysis

Antibody	Clone	Supplier (cat#)
Anti-CD45	30-F11	BD Biosciences (563891)
Anti-CD3	145-2C11	Biolegend (100312)
Anti-CD19	1D3	BD Biosciences (557399)
Anti-CD4	RM4-5	BD Biosciences (558107)
Anti-CD8a	53-6.7	BD Biosciences (100766)
Anti-CD11b	M1/70	Biolegend (101217)
Anti-Ly6G	1A8	Biolegend (127615)
Anti-CD64	X54-5/7.1	Biolegend (139303)
Anti-MHCII	M5/114.15.2	Biolegend (107613)
Anti-CCR2	SA203G11	Biolegend (150605)
Anti-Ly6C	AL-21	BD Biosciences (562727)
7AAD		Biolegend (420404)
Fixable viability dye eFluor 780		eBioscience (65-0865-14)

Supplemental Table S4: Echocardiographic measurements calculated from Simpson's Mode for control and iCMp38αKO hearts over time course (baseline, 12, 24 and 48 hours AngII treatment). Data are mean ± SD. Baseline values showed no statistically significant differences. Data were analyzed using two-way ANOVA followed by Bonferoni post-test. Comparison to control: * $p<0.05$, ** $p<0.01$, *** $p<0.001$, Comparison to baseline: °$p<0.05$, °°$p<0.01$, °°°$p<0.001$

**Control**		**Baseline**		**12h AngII**			**24h AngII**			**48h AngII**		
Parameter		**Mean**	**SD**	**Mean**	**SD**		**Mean**	**SD**		**Mean**	**SD**	
Cardiac Output	ml/min	27.0	4.3	21.3	2.4	°	23.7	2.3		22.8	8.0	
Ejection Fraction	%	59.8	4.7	41.5	12.0	°°°	46.9	3.9	°°	49.1	12.0	°
Fractional Area Change	%	48.5	6.6	31.5	16.0	°°°	27.2	2.3	°°°	31.4	13.4	°°°
Fractional Shortening	%	12.0	3.8	7.4	1.7		11.7	3.2		15.0	7.3	
Stroke Volume	ul	46.8	6.7	34.4	3.3	°°	39.3	4.2		38.0	13.1	°
End Diastolic Volume	ul	78.7	12.1	88.6	25.5		84.1	7.9		76.6	10.6	
End Systolic Volume	ul	31.9	7.3	54.2	22.3	°°	44.8	6.1		38.6	9.4	
Heart Rate	bpm	574	19.5	617	30.8	°	591	21.7		588	40.2	
**iCMp38αKO**		**Baseline**		**12h AngII**			**24h AngII**			**48h AngII**		
Parameter		**Mean**	**SD**	**Mean**	**SD**		**Mean**	**SD**		**Mean**	**SD**	
Cardiac Output	ml/min	27.4	4.6	19.2	3.2	°°	18.3	3.4	°°°	18.4	5.2	°°°
Ejection Fraction	%	59.6	8.1	33.3	5.6	°°°	24.1	5.3	***<br>°°°	29.0	7.6	***<br>°°°
Fractional Area Change	%	48.4	8.2	20.8	6.6	°°°	13.4	6.5	°°°	15.0	7.0	**<br>°°°
Fractional Shortening	%	13.3	4.8	7.9	0.9	°	5.3	2.1	°°°	7.0	3.9	**<br>°°
Stroke Volume	ul	48.6	8.5	30.7	4.4	°°°	30.5	4.3	°°°	31.3	6.8	°°°
End Diastolic Volume	ul	83.8	22.5	92.5	5.1		129.2	18.7	***<br>°°°	109.7	14.6	**<br>°°
End Systolic Volume	ul	35.3	15.8	61.8	8.0	°°°	98.7	19.3	***<br>°°°	78.4	16.5	***<br>°°°
Heart Rate	bpm	561.4	24.3	620.8	27.2	°°°	580.6	21.5		570	41.5	

Supplemental Table S5: Atglistatin treatment: Cardiac function calculated from Simpson's mode of iCMp38αKO and control mice at baseline (day -2) and before AngII administration (day 0). Statistical analysis was performed using two-way ANOVA with Bonferroni's multiple comparisons test to compare the two timepoints within each group. No statistically significant differences.

		**Control day -2**		**Control day 0**		**KO day -2**		**KO day 0**	
Parameter	Units	**Mean**	**SD**	**Mean**	**SD**	**Mean**	**SD**	**Mean**	**SD**
Cardiac Output	ml/min	30.73	4.04	27.38	4.27	27.88	4.51	28.37	2.47
Ejection Fraction	%	60.41	5.54	58.67	2.53	56.27	7.57	60.54	5.29
Fractional Area Change	%	53.84	9.36	49.40	4.25	46.32	8.81	50.43	9.61
Fractional Shortening	%	15.96	3.68	14.64	4.13	15.19	2.81	18.19	5.75
Stroke Volume	µL	54.23	8.00	51.32	6.52	49.35	7.26	52.42	3.82
End Diastolic Volume	µL	90.68	16.55	87.45	10.20	88.95	15.70	87.26	10.40
End Systolic Volume	µL	36.45	10.59	36.13	4.73	39.60	12.46	34.84	8.14
HR	bpm	569.50	27.95	542.75	28.38	565.50	31.58	551.70	30.46

Supplemental Table S6: Cardiac function calculated from Simpson's mode of iCMp38αKO and control mice before granulocyte depletion. No statistically significant differences were found between control and KO.

		**Control Baseline**		**KO Baseline**	
Parameter	Units	**Mean**	**SD**	**Mean**	**SD**
Cardiac Output	ml/min	28.37	2.86	31.36	5.55
Ejection Fraction	%	62.76	8.39	59.75	6.57
Fractional Area Change	%	51.56	8.92	47.89	7.20
Fractional Shortening	%	14.96	4.50	13.38	4.26
Stroke Volume	ul	51.10	6.15	56.40	10.25
End Diastolic Volume	ul	82.64	14.71	95.70	21.01
End Systolic Volume	ul	31.54	11.91	39.30	13.36
HR	bpm	568	50	562	18.4

A

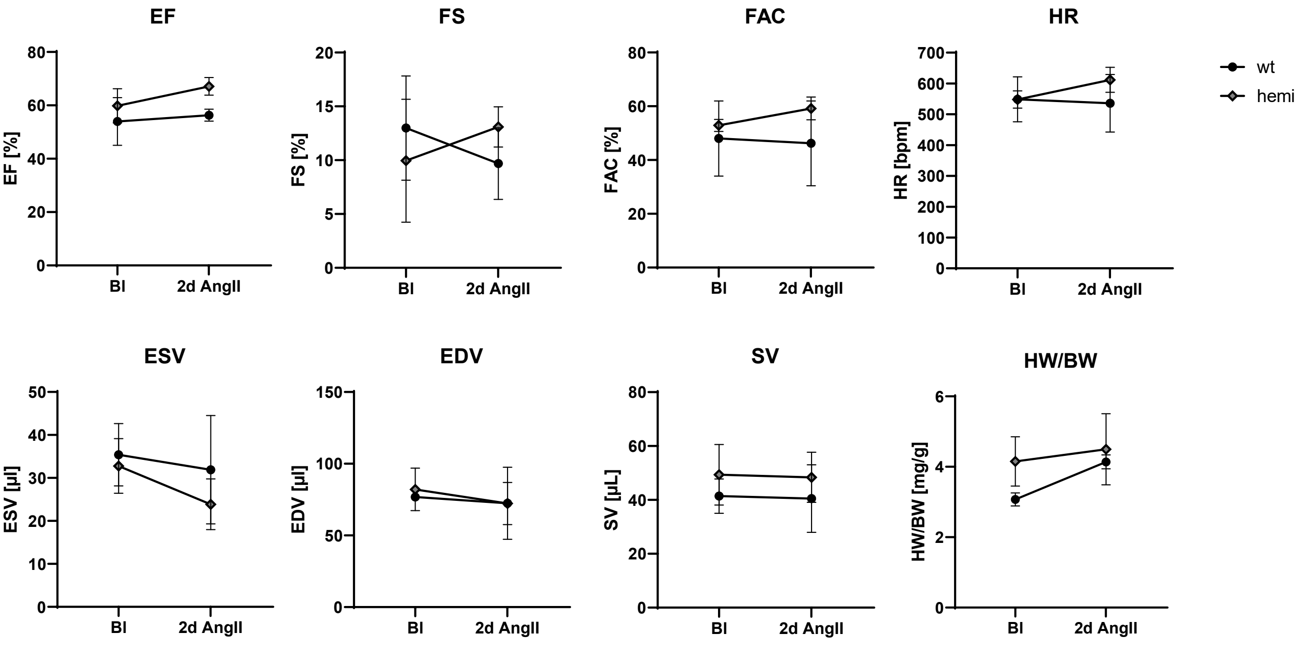

Supplemental Fig. S1.: Cardiac function and volumes, heart rate and heart weight/body weight ratio in MerCreMer hemizygous male mice (hemi) and wildtype (wt) littermates at baseline and after 2d AngII treatment. No statistical significant differences were detected between the two groups at both time points. Data are presented as mean ± SD, hemi n=4, wt n=2, two-way ANOVA followed by Bonferoni's multiple comparisons test, compared were hemi vs wt at baseline and 2d AngII.

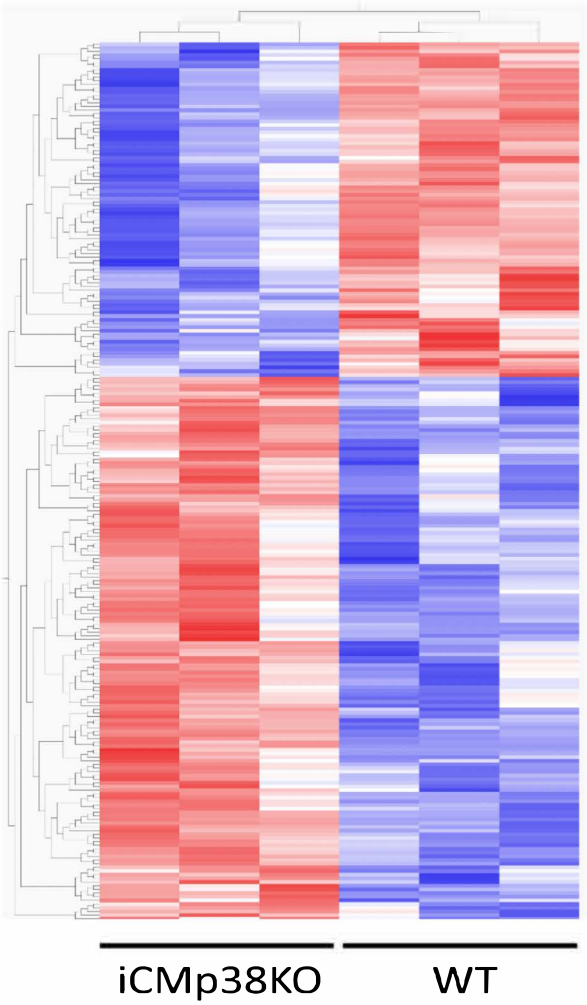

Pathway	-log P-value	Z-score	Down-regulated	No change	Up-regulated	No overlap with dataset	Molecules
**Oxidative Phosphory-lation**	8,4	-3,32	11/110 (10%)	0/110 (0%)	0/110 (0%)	99/110 (90%)	ATP5F1D, COX6A1,COX8A, NDUFA11,NDUFA2,NDUFA5,NDUFB10,NDUFB7,NDUFS8,NDUFV3, UQCRQ
**Superpathway of Inositol Phosphate Compounds**	1,75	2,45	0/232 (0%)	0/232 (0%)	6/232 (3%)	226/232 (97%)	IP6K3,MTMR9, PAWR,PIK3R1,PPIP5K1,PTPN11
**3-phospho-inositide Biosynthesis**	1,46	2,24	0/202 (0%)	0/202 (0%)	5/202 (2%)	197/202 (98%)	MTMR9,PAWR, PIK3R1,PPIP5K1, PTPN11

Supplemental Fig. S2: A:Hierarchial clustering of differentially expressed genes in hearts of control (WT) and iCMp38KO mice after 12h of AngII treatment. ($p<0.05$, fold change>1.3). B: Result of pathway analysis using Ingenuity Pathway Analysis software (IPA[(R)], Qiagen Inc.). Only pathways with -log P-value <1.3 (0.05) and an absolute activation Z-score>2 were considered to be affected. Only a small number of genes assembled under the respective term was found to be differentially expressed.

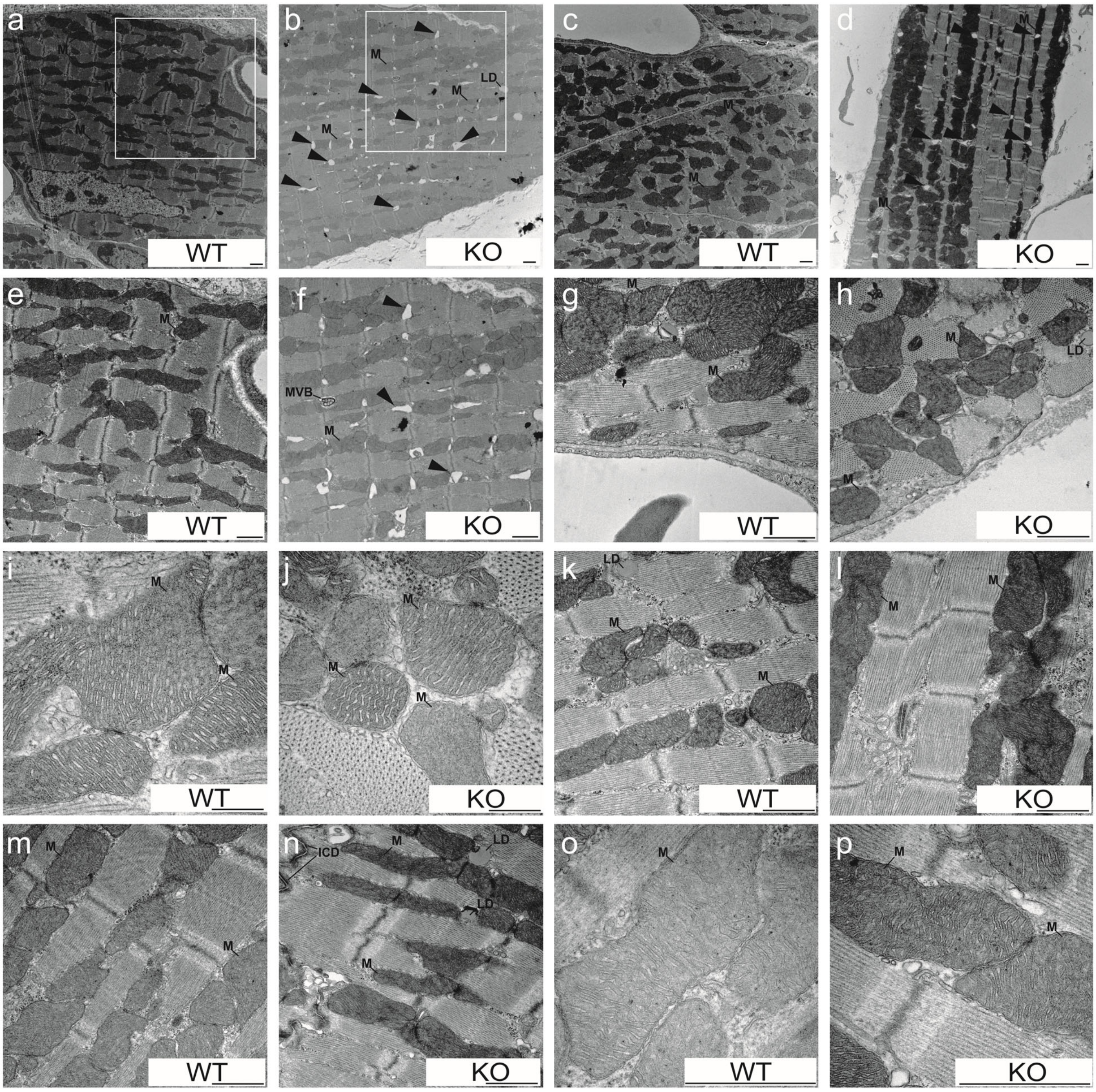

Supplemental Fig. S3: Transmission electron microsopy of control (WT) and iCMp38KO (KO) hearts. Tissue samples from three regions of the heart were chemically fixed by 1.2% glutaraldehyde and 1% paraformaldehyde, and ultrathin sections were analysed by standard transmission electron microscopy. a, b, e, f sections from LV free wall ; c, d, g, h, i, j, k, l, sections from anterior wall ; m, n, o, p sections from interventricular septum . MVB, multivesicular body; LD, lipid droplet; M, mitochondrium; ICD, intercalated disc; arrow heads show enlarged lumen at T-tubule cross sections which were observed in iCMp38KO sections only. T-tubule swelling has been observed also in human DCM. Scale bars, 1 μm.

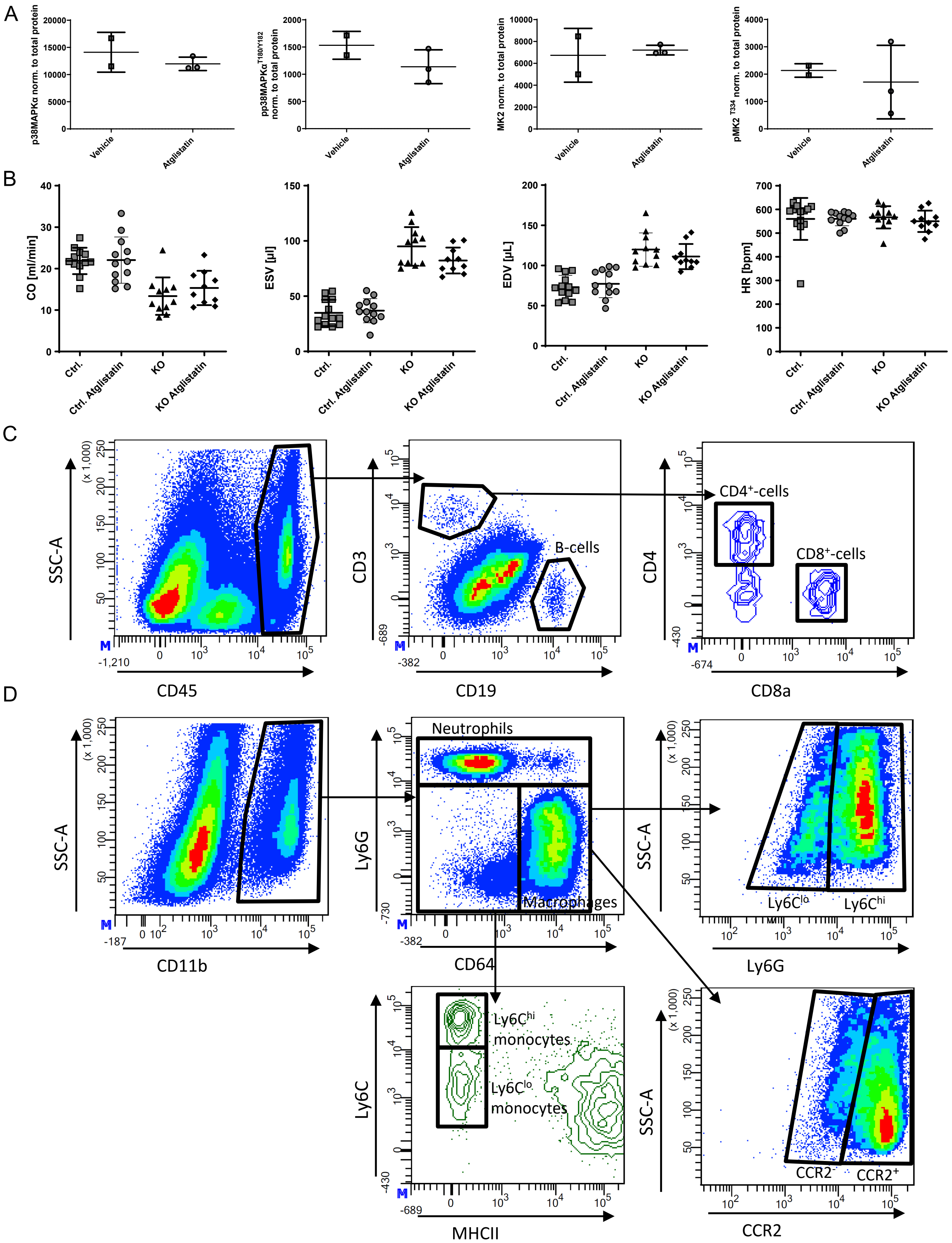

Supplemental Fig. S4.: A) Protein expression of p38MAPKα, pp38MAPKα, MK2 and pMK2 in vehicle and Atglistatin treated control animals B) Cardiac output (CO), end systolic (ESV) and end diastolic volume (EDV) and heart rate (bmp) of iCMp38α control and KO mice after 2 days of Angiotensin II either treated with Atglistatin or vehicle. B) Gating scheme for flow cyometric analysis of B-lymphocytes (CD45^{+}CD19^{+}) and CD4^{+}, and CD8^{+} T-lymphocytes (CD45^{+}CD3^{+}) in heart cell preparations. C) Gating scheme for flow cytometric analysis of neutrophils (CD11b^{+}Ly6G^{+}), Ly6C^{hi} and Ly6C^{lo} monocytes (CD11b^{+}Ly6G^{+}CD64^{-}MHCII^{-}), and CCR2^{+}, CCR2^{-}, Ly6C^{hi} and Ly6C^{lo} macrophages (CD11b^{+}Ly6G^{+}CD64^{+}) in heart cell preparations.

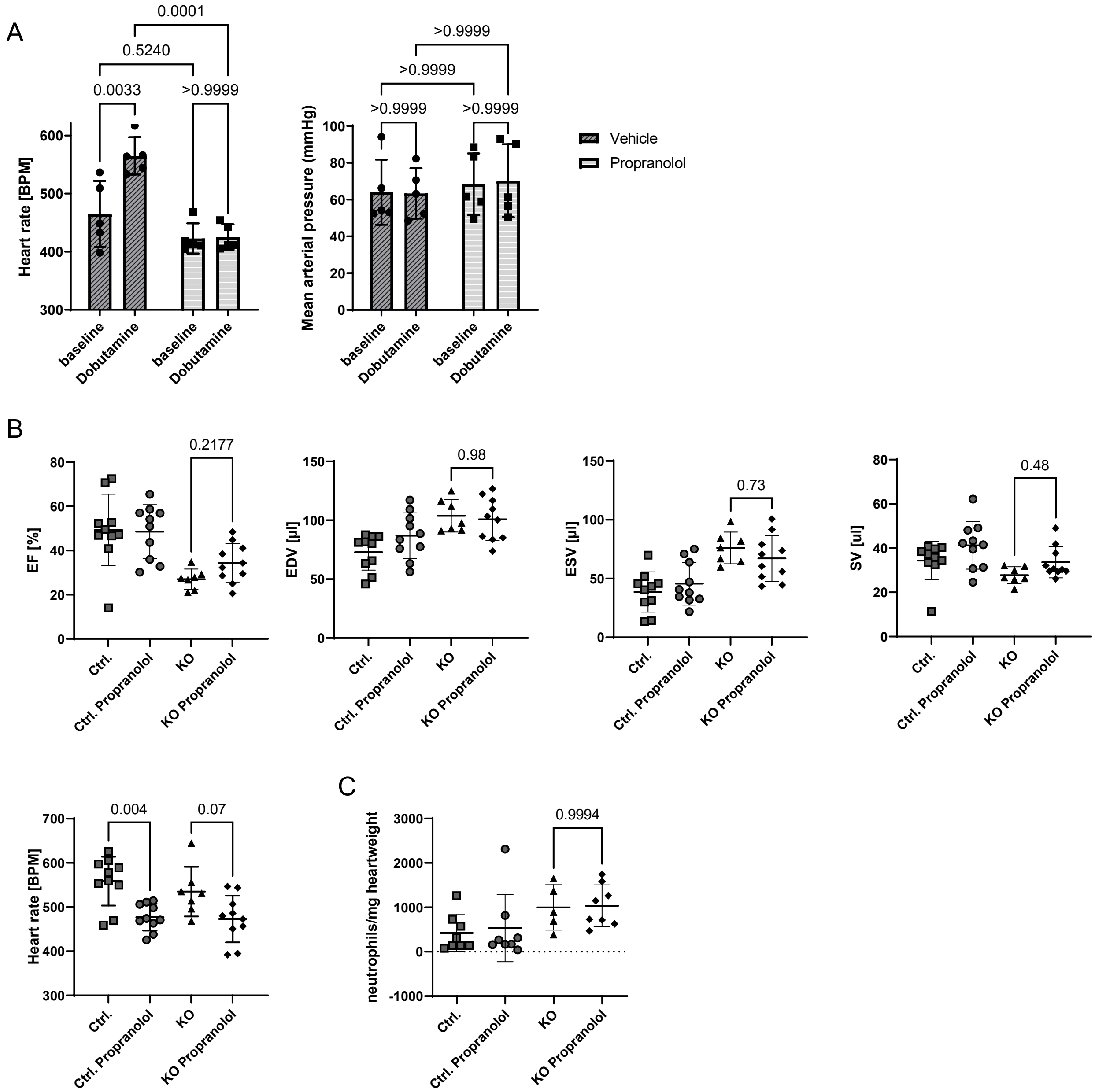

Supplemental Fig. S5: A) Heart rate and mean arterial pressure in vehicle and propranolol treated wildtype animals at baseline and after dobutamine treatment. Propranolol prevents increase in heart rate due to dobutamine treatment and does not lower blood pressure. Two-way ANOVA with Bonferroni's multiple comparisons test was used. B) Ejection fraction (EF), end diastolic (EDV), end systolic (ESV), stroke volume (SV) and heart rate of iCMp38α control and KO mice after 2 days of Angiotensin II either treated with propranolol or vehicle. One-way ANOVA with Tukey's multiple comparisons test was used. C) Neutrophil number/mg heart weight in hearts of iCMp38α control and KO mice after 2 days of Angiotensin II either treated with propranolol or vehicle. One-way ANOVA with Tukey's multiple comparisons test was used. All data are mean ± SD.

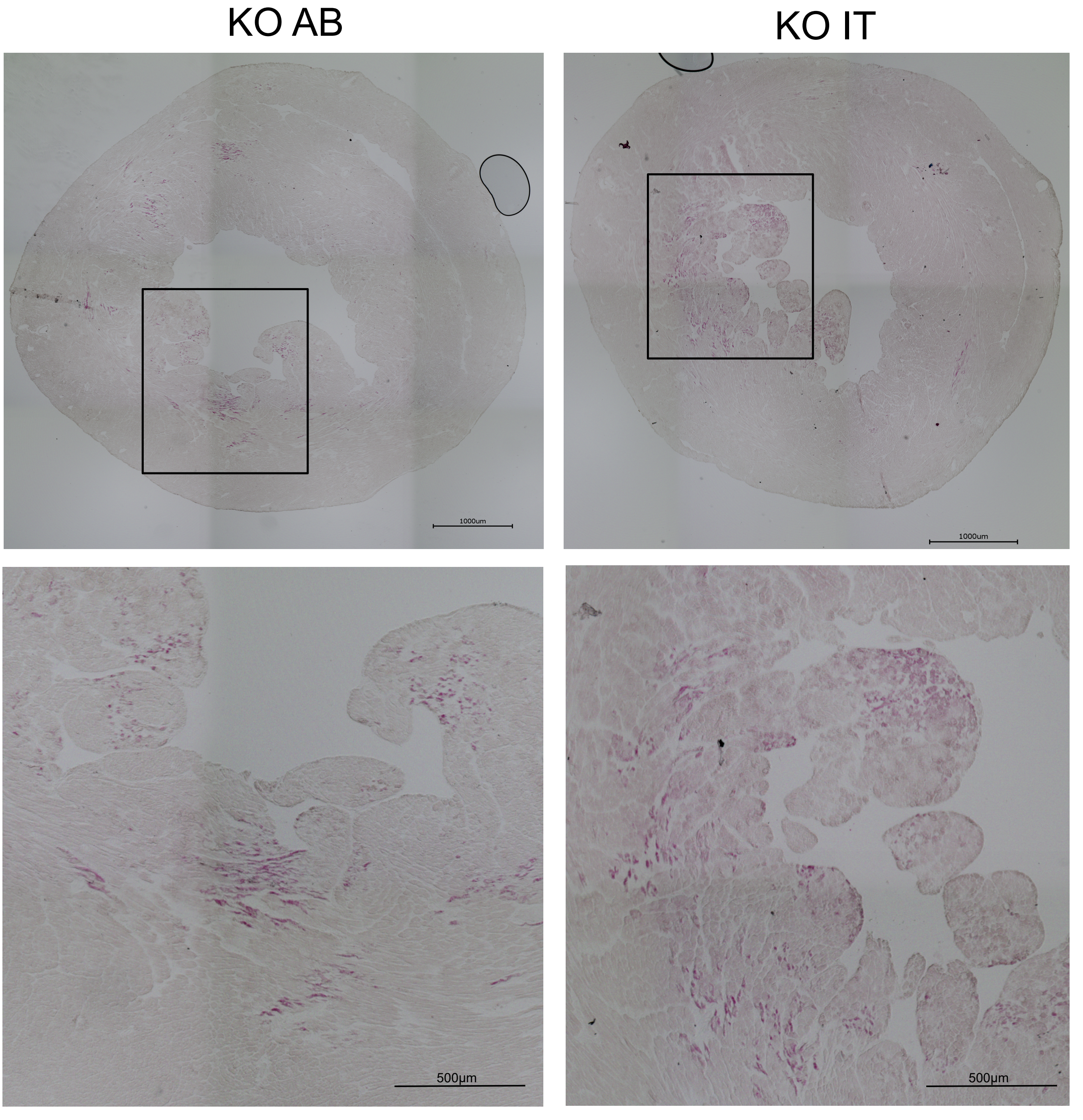

Supplemental Fig. S6: Sudan Red 7B Staining of iCMp38αKO hearts after 48h AngII either treated with anti-Ly6G antibody (AB) or isotype control (IT). Lower panels show corresponding magnifications of indicated areas.
